# Integrative analysis of Paneth cell proteomic and transcriptomic data from intestinal organoids reveals functional processes dependent on autophagy

**DOI:** 10.1242/dmm.037069

**Published:** 2019-03-18

**Authors:** Emily J. Jones, Zoe J. Matthews, Lejla Gul, Padhmanand Sudhakar, Agatha Treveil, Devina Divekar, Jasmine Buck, Tomasz Wrzesinski, Matthew Jefferson, Stuart D. Armstrong, Lindsay J. Hall, Alastair J. M. Watson, Simon R. Carding, Wilfried Haerty, Federica Di Palma, Ulrike Mayer, Penny P. Powell, Isabelle Hautefort, Tom Wileman, Tamas Korcsmaros

**Affiliations:** 1 Earlham Institute, Norwich Research Park, Norwich NR4 7UZ, UK; 2 Quadram Institute, Norwich Research Park, Norwich NR4 7UA, UK; 3 Norwich Medical School, University of East Anglia, Norwich NR4 7TJ, UK; 4 National Institute of Health Research, University of Liverpool, Liverpool L3 5RF, UK; 5 School of Biological Sciences, University of East Anglia, Norwich NR4 7TJ, UK

**Keywords:** Paneth cells, Atg16l1, Intestinal organoids, Quantitative proteomics, Selective autophagy

## Abstract

Paneth cells are key epithelial cells that provide an antimicrobial barrier and maintain integrity of the small-intestinal stem cell niche. Paneth cell abnormalities are unfortunately detrimental to gut health and are often associated with digestive pathologies such as Crohn's disease or infections. Similar alterations are observed in individuals with impaired autophagy, a process that recycles cellular components. The direct effect of autophagy impairment on Paneth cells has not been analysed. To investigate this, we generated a mouse model lacking *Atg16l1* specifically in intestinal epithelial cells, making these cells impaired in autophagy. Using three-dimensional intestinal organoids enriched for Paneth cells, we compared the proteomic profiles of wild-type and autophagy-impaired organoids. We used an integrated computational approach combining protein-protein interaction networks, autophagy-targeted proteins and functional information to identify the mechanistic link between autophagy impairment and disrupted pathways. Of the 284 altered proteins, 198 (70%) were more abundant in autophagy-impaired organoids, suggesting reduced protein degradation. Interestingly, these differentially abundant proteins comprised 116 proteins (41%) that are predicted targets of the selective autophagy proteins p62, LC3 and ATG16L1. Our integrative analysis revealed autophagy-mediated mechanisms that degrade key proteins in Paneth cell functions, such as exocytosis, apoptosis and DNA damage repair. Transcriptomic profiling of additional organoids confirmed that 90% of the observed changes upon autophagy alteration have effects at the protein level, not on gene expression. We performed further validation experiments showing differential lysozyme secretion, confirming our computationally inferred downregulation of exocytosis. Our observations could explain how protein-level alterations affect Paneth cell homeostatic functions upon autophagy impairment.

This article has an associated First Person interview with the joint first authors of the paper.

## Abstract (precis)

**Editor's choice:** Using an integrative approach encompassing intestinal organoid culture, proteomics, transcriptomics and protein-protein interaction networks, we list Paneth cell functions dependent on autophagy.

## INTRODUCTION

Paneth cells, located at the bottom of the crypts of Lieberkühn in the small intestine, secrete various types of antimicrobial compounds (e.g. lysozyme, defensins) to regulate the microbial composition of the intestine, as well as growth factors that maintain the crypt-associated stem cell population (Bevins and Salzman, 2011). The secretory activity of Paneth cells strongly relies on pathways to release proteins such as antimicrobials into the lumen as one of the gut barrier functions contributing to intestinal homeostasis (Bel et al., 2017; Liu et al., 2013). Conventional protein secretion involves trafficking through the endoplasmic reticulum (ER) and Golgi (Farquhar and Palade, 1981; Viotti, 2016). Paneth cell defects such as altered granule morphology and increased susceptibility to ER stress are seen in mouse models in which autophagy is lost from intestinal epithelial cells (Liu et al., 2013; Wileman, 2013).

Autophagy is a pivotal recycling process that sequesters cytoplasmic misfolded proteins or damaged organelles as well as clearing the cytosol from invading pathogens. These targets are captured in double-membrane vesicles called autophagosomes that are subsequently delivered for degradation to lysosomal compartments (Deretic et al., 2013; Glick et al., 2010; Todde et al., 2009; Wileman, 2013). Although initially considered as a non-selective process elicited upon starvation, stress or infection, recent studies have indicated that the cargoes of autophagy, be it organelles (such as mitochondria, peroxisomes, ribosomes, ER), pathogens or protein aggregates, are recognised in a very selective manner, termed selective autophagy (Fimia et al., 2013; Zaffagnini and Martens, 2016). Sequestration of selective-autophagy targets follows recognition by specific cargo receptors and involves the Atg12-Atg5-Atg16 complex, instrumental in the early stages of autophagosome biogenesis by determining the site of LC3 lipidation (Fujita et al., 2008). Through LIR (LC3-interacting region) motifs, the lipidated LC3 adaptor not only targets various cargoes for sequestration but also recruits multiple autophagy receptor proteins such as p62, NDP52, NBR1, NIX and optineurin. Cargo recognition by the autophagy receptors happens generally via ubiquitin-dependent or ubiquitin-independent mechanisms (Khaminets et al., 2016). The autophagy receptors bridge their cargoes (which are specifically targeted by the presence of receptor-recognition motifs and degradation signals) with the autophagosomal membrane (Stolz et al., 2014). These events eventually result in cargo engulfment by the autophagosome, which then fuses with the lysosome to form the autophagolysosome, in which the contents are degraded by lysosomal enzymes (Glick et al., 2010; Todde et al., 2009). In addition to its recycling role, autophagy is involved in the Paneth cell response to microbial exposure. Upon microbial challenge, lysozyme secretion by Paneth cells is conducted through the diversion of degradative autophagy towards a secretory process, named the secretory autophagy pathway (Bel et al., 2017; Kimura et al., 2017), although various autophagy-independent secretory pathways have also been reported (Barlowe and Miller, 2013).

Alterations in secretory autophagy have been associated with many intestinal diseases. Severe gut pathologies, such as Crohn's disease (CD) – an inflammatory bowel disease (IBD) – or food-borne pathogen infections (e.g. salmonellosis), are associated with Paneth cell dysfunctions, including disrupted antimicrobial production, as observed in chronic inflammatory and infectious diseases (Liu et al., 2016; Martinez Rodriguez et al., 2012; Perminow et al., 2010; Salzman et al., 2003; Wehkamp et al., 2005). Genome-wide association studies (GWAS) have identified mutations in autophagy-related genes – in particular, mutation in the key autophagy gene, *ATG16L1* – that result in granule exocytosis abnormalities in Paneth cells, with a negative effect on autophagy-mediated defence against bacterial pathogens (Cadwell et al., 2008; Lassen et al., 2014; Perminow et al., 2010; Wehkamp et al., 2005). Owing to its critical function in the autophagy machinery, ATG16L1 is required for the proper functioning of autophagy in general (Kuballa et al., 2008; Mizushima et al., 2003) and in various intestinal cell types, including Paneth cells (Cadwell et al., 2008; Patel et al., 2013). In Paneth cells of mice harbouring mutations in key autophagy genes, such as *Atg7* or *Atg16l1*, lysozyme levels were decreased, granule size reduced and exocytosis abnormal, compared with wild-type (WT) mice (Cadwell et al., 2008; Conway et al., 2013; Lassen et al., 2014; Wittkopf et al., 2012). Specific mutations in *Atg16l1*, such as T300A, affect the activity of *Atg16l1* due to the gain of a caspase-3 cleavage site without compromising the protein architecture (Salem et al., 2015). Even though the critical role of ATG16L1 in modulating autophagy in Paneth cells is known, the exact molecular mechanisms and cellular processes affected by autophagy impairment remain to be elucidated.

**Fig. 1.**
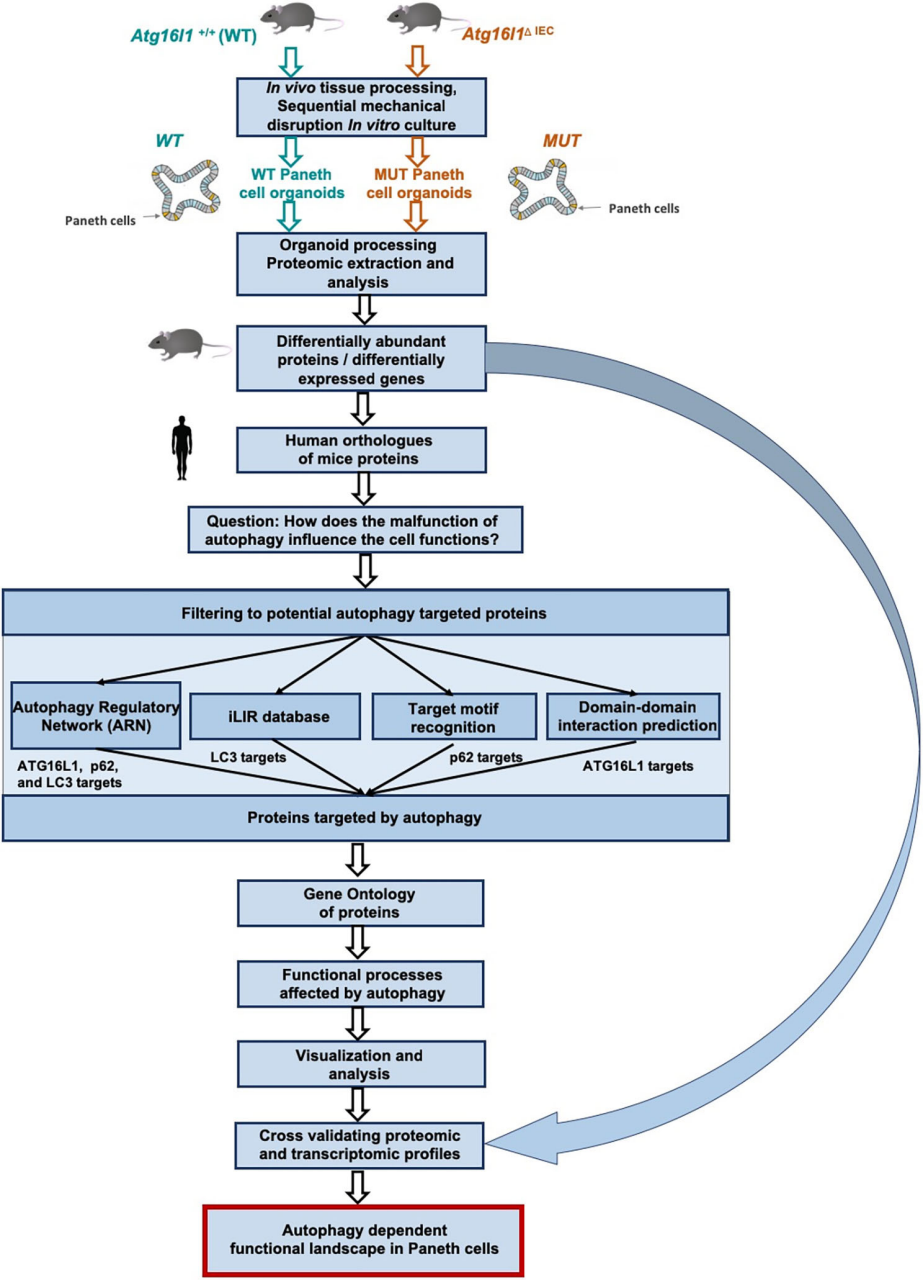
**A schematic representation of the workflow to determine the functional effects of autophagy impairment in *Atg16l11*^ΔIEC^ in Paneth cell organoids.** Three biological replicates were generated for each condition and genotype tested, and for both the proteomic and transcriptomic type of profiling.

In this study, we use the small-intestinal organoid culture model, which reproduces crypt-like and villus-like domains characteristic of intestinal morphology, recapitulating many functions of the small bowel. Intestinal organoids contain specialised cell types, such as Paneth cells, that cannot be examined in cell lines, making them a unique model system to analyse Paneth cell proteins and functions (Sato et al., 2009). To increase the usefulness of the organoid model, we enrich both WT and autophagy-impaired organoids for Paneth cells by directing the lineage of organoid differentiation (Luu et al., 2018). In our previous report we show that drug-treated organoids recapitulate important features of the *in vivo* gut environment, demonstrating that they can serve as useful models for the investigation of normal and disease processes in the intestine. We compared mass-spectrometry data with histology data contained within the Human Protein Atlas and identified putative novel markers for goblet and Paneth cells (Luu et al., 2018). In this study, we analyse the quantitative proteome of Paneth-cell-enriched small-intestinal organoids specifically lacking *Atg16l1* in intestinal epithelial cells, and compare it to the proteomic profile of WT Paneth-cell-enriched organoids. Given the known defects of autophagy in inflammatory disorders, the major autophagy impairment due to the loss of Atg16l1 could be considered as an extreme disease model. In order to understand the possible mechanisms by which autophagy impairment could modulate the abundance of proteins in key epithelial cell functions, we establish an *in silico* workflow (Fig. 1) combining several computational approaches, including protein-protein interaction networks, interaction evidence incorporating protein targeting by selective autophagy and information on functional processes. Using this integrative approach, we show that proteins with altered abundances in the autophagy-impaired Paneth-cell-enriched organoids could be substrates of selective autophagy and could be targeted by autophagy, resulting in their degradation. Our integrative approach pointed out several autophagy-dependent cellular processes as well as novel mechanisms in which autophagy was influencing those processes. Using the transcriptomic profiling of the WT and autophagy-impaired organoids, we validate that the proteomic changes are due to protein-level alterations and not due to gene expression changes. Importantly, we also confirm that autophagy dysfunction alters several cellular processes, such as cellular exocytosis, which was downregulated in autophagy-impaired organoids and is known to be deleteriously altered in patients with an inflamed digestive tract (e.g. CD patients). Taken together, our observations, based on a model of autophagy impairment in Paneth cells, provide a mechanistic explanation of Paneth cell dysfunction due to autophagy impairment. The demonstrated involvement of novel autophagy-dependent processes in Paneth cells extends our understanding of disorders related to autophagy dysfunction. Furthermore, it opens the door for the development of new and/or supplementary therapeutic interventions for digestive pathologies triggered or exacerbated upon autophagy deficiency.

## RESULTS

### Paneth-cell-enriched *Atg16l1*^ΔIEC^ organoids are a valid model to study the role of autophagy in intestinal epithelial homeostasis

**Fig. 2.**
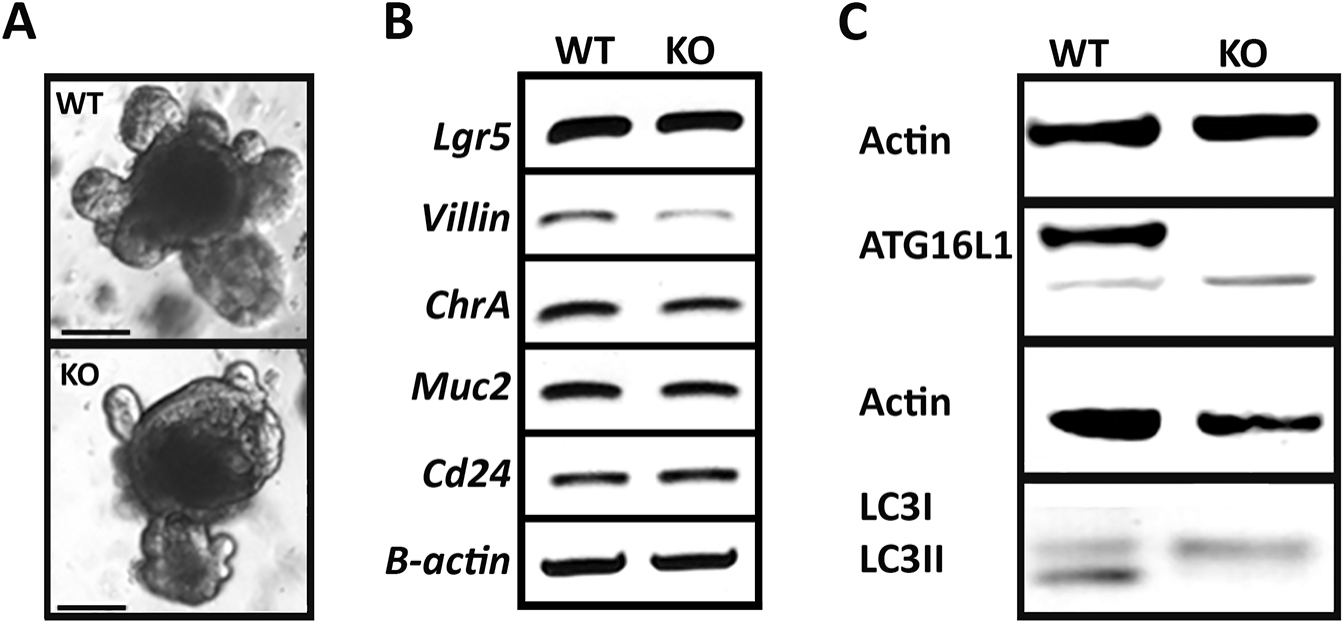
***Atg16l11*^ΔIEC^ organoids contain the same intestinal epithelial cell types as WT organoids but lack Atg16l1 and LC3II both in the transcriptional and protein levels (*n*=3).** (A) Brightfield image of control (WT) and *Atg16l1*^ΔIEC^ (KO) organoids after 7 days of growth. Magnification: 10×; scale bars: 100 µm. (B) Cell-type-specific RT-PCR amplification of cDNAs in WT and *Atg16l1*^ΔIEC^ (KO) organoids. β-actin was used as a housekeeping gene. (C) Western blots using anti-ATG16L1 and -LC3 antibodies detected Atg16l1 and LC3II in control organoids but *Atg16l1*^ΔIEC^ (KO) organoids were deficient in Atg16l1 and LC3II.

We have generated a mouse model that lacks *Atg16l1* specifically in intestinal epithelial cells (*Atg16l1*^ΔIEC^) and have used self-organising *in vitro* organoid cultures generated from small-intestinal crypts (Sato et al., 2009) to assess the impact of autophagy on Paneth cell functions. As expected, normally differentiated organoids from both WT and *Atg16l1*^ΔIEC^ mice included viable budding crypts that expanded from a core organoid (Fig. 2A). Detection of mRNA transcripts by linear reverse transcription PCR (RT-PCR) for *Lgr5*, *ChgA*, *Muc2* and *Cd24* cDNAs along with the housekeeping β-actin gene revealed that *Atg16l1*^ΔIEC^ organoids expressed markers for stem cells, enteroendocrine cells, goblet cells and Paneth cells at similar levels as WT organoids (Fig. 2B), confirming similar differentiation expression levels in both genotypes of important cell types found in the *in vivo* small-intestinal epithelium. We observed that the villin transcript shows a slight reduction in the knockout (KO) organoids compared with the WT ones, but remains indicative of the presence of enterocytes in both organoid models. In particular, we noted that the level of the Paneth cell marker CD24 was similar between WT and *Atg16l1*^ΔIEC^ organoids (Fig. 2B), suggesting that the number of Paneth cells was similar in both genetic backgrounds.

To increase the technical feasibility of investigating the dependency of Paneth cells on autophagy, organoids were further enriched for Paneth cells using a well-established and published protocol, presented in detail in the Materials and Methods (Nakanishi et al., 2016; Yin et al., 2014). We confirmed Paneth cell enrichment using two complementary approaches. First, we observed the transcriptomic profiles generated from WT and *Atg16l1*^ΔIEC^ genotypes. For each genotype, we generated a list of differentially expressed genes by comparing Paneth-cell-enriched to normally differentiated organoids. Genes previously identified as Paneth cell markers were observed in these lists of differentially expressed genes (Haber et al., 2017). Paneth cell markers were significantly enriched in the gene expression dataset generated from WT organoids (56/83 markers present, hypergeometric test, *P*=5.1×10^–57^) and from *Atg16l1*^ΔIEC^ organoids (58/83 markers present, hypergeometric test, *P*=3.2×10^–14^), suggesting that Paneth cells were successfully enriched through the applied enrichment protocol. Second, we compared the proteomic profiles of normally differentiated intestinal organoids with that of Paneth-cell-enriched organoids, focussing on organoids of WT and *Atg16l1*^ΔIEC^ genotypes (Tables S3 and S4). Using a similar workflow as for the subsequent comparison between the WT and *Atg16l1*-deficient Paneth-cell-enriched organoids (Fig. 1), we observed proteins with significantly different abundance between normally differentiated and Paneth-cell-enriched organoids. We observed that Paneth-cell-specific processes were altered upon impaired autophagy in the enriched organoids. Proteins related to exocytosis, proteasome-ubiquitin-system-related processes, immune response and apoptosis were differentially abundant in Paneth-cell-enriched organoids. Overall, the two approaches support the conclusion that the organoids used in this study were enriched with Paneth cells in both WT and *Atg16l1*^ΔIEC^ organoids.

We then sought confirmation that autophagy was affected in *Atg16l1*^ΔIEC^ organoids. Consistent with the intestinal epithelial-cell-specific *Atg16l1* deficiency, western blot analysis confirmed that *Atg16l1*^ΔIEC^ KO organoids were deficient in the Atg16l1 protein. Atg16l1 was detected in the WT organoid samples at 68 kDa, but not in *Atg16l1*^ΔIEC^ organoids even though a non-specific band is seen with the used antibody at 66 kDa. In addition, we also observed LC3I to LC3II conversion in WT but not in the *Atg16l1*^ΔIEC^ organoids, thus indicating that *Atg16l1* deletion leads to autophagy deficiency. As observed in previous studies, lack of *Atg16l1* resulted in impairment of autophagy as corroborated by reduced levels of LC3II (Fig. 2C; Cadwell et al., 2008; Patel et al., 2013). Together, these observations validate *Atg16l1*^ΔIEC^ organoids as a robust model for investigating the impairment of autophagy in epithelial homeostasis.

### Alteration in the proteomic abundance profiles upon autophagy impairment

**Fig. 3.**
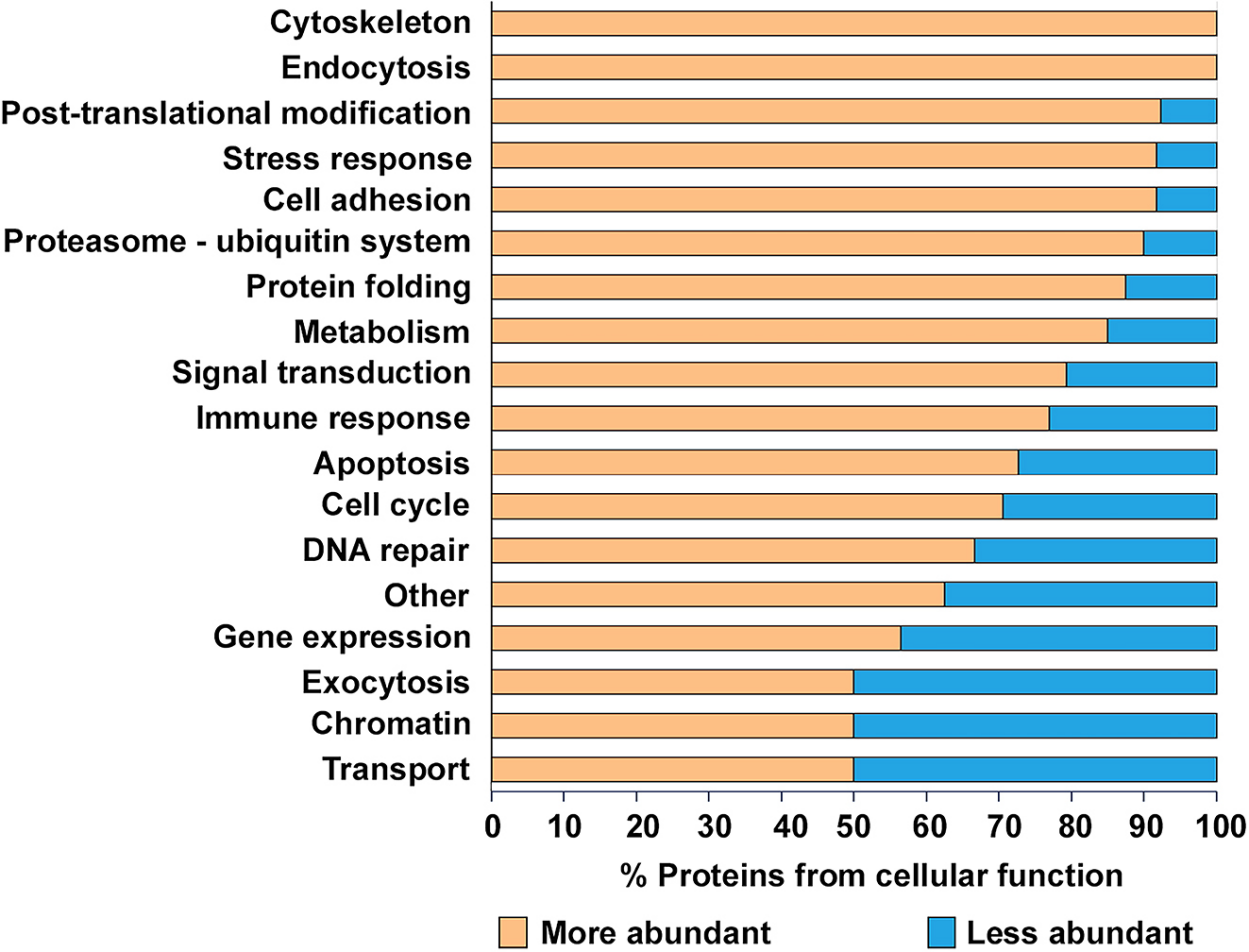
**Percentage of higher- and lower-abundance proteins in different cellular functions.** Proteins with higher abundance are marked with orange and lower-abundance proteins with a blue background. The ‘Other’ category contains all of the proteins that did not fit into other functional groups.

In order to determine the functional significance of the *Atg16l1* deficiency in Paneth cells, we established an integrated workflow (Fig. 1) combining computational approaches to integrate and interpret the experimental readouts. We measured the protein levels in Paneth-cell-enriched organoids derived from WT mice and mice harbouring the *Atg16l1* deficiency, with three biological replicates generated per condition tested. Our proteomic experiments detected 283 mouse proteins corresponding to 284 human orthologue proteins with altered abundances at the cut-offs used [*P*<0.05, absolute relative fold-change (FC) ≥2, number of unique peptides ≥2] (Table S5). Our initial functional analysis showed that proteins with altered abundance were related to at least 18 functional processes (Fig. 3), and that the majority (70%) of all of these proteins were detected at levels twice greater than those found in WT organoids (Table S6), suggesting that the observed higher abundance could be a due to autophagy impairment.

### Proteins potentially targeted by selective autophagy have altered abundances in *Atg16l1*^ΔIEC^ Paneth cell organoids

**Fig. 4.**
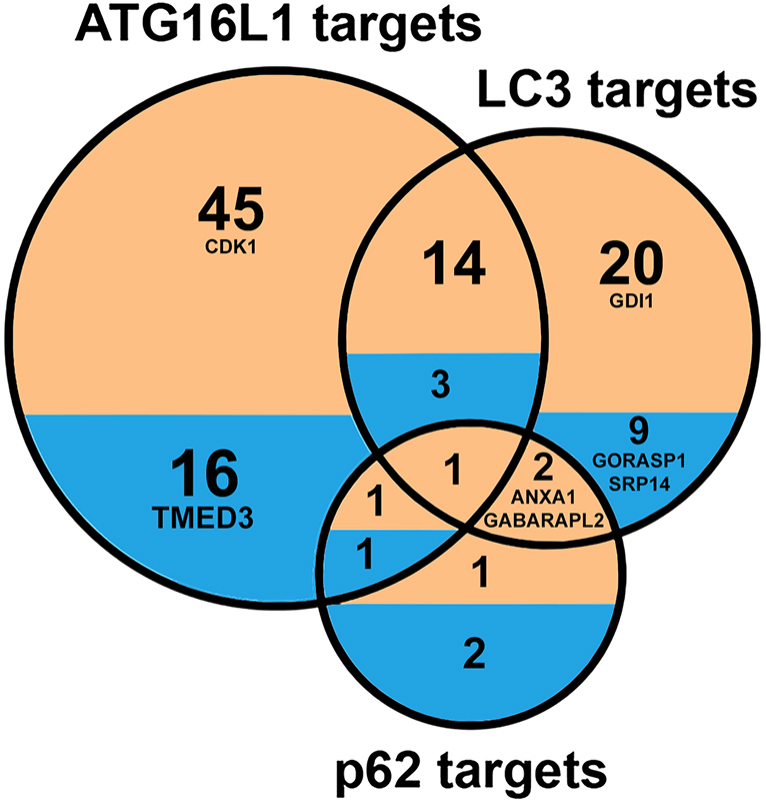
**Overlap between the proteins with altered abundances in *Atg16l1*^ΔIEC^** **Paneth cell organoids.** The diagram is restricted to proteins potentially targeted by each of the three selective autophagy-mediating proteins – p62, LC3 and Atg16l1 – under normal circumstances without any defects in autophagy.

Since the primary role of autophagy is to identify, target and recycle damaged proteins, altered protein levels in *Atg16l1*^▵IEC^ Paneth-cell-enriched organoids reflect the possible effect of disrupted autophagy. To determine whether autophagy directly or indirectly affects the proteins that are differentially abundant in the autophagy-impaired background, we compared the altered proteins with the target proteins of known selective autophagy receptors and adaptors, such as p62, LC3 and Atg16l1 (Table 1). This network analysis and the subsequent functional investigations were performed using human data (by inferring the human orthologues of the differentially abundant mouse proteins) due to increased data availability on human networks/ontologies and thereby an increased coverage. By incorporating information about the binding partners (using experimental evidence and structure-based predictions) of the human orthologues, we identified the autophagy-targeting proteins that could potentially target the altered proteins in Paneth cell organoids. In total, 116 proteins (41%; *P*=0.049) with altered abundance in autophagy-impaired organoids and, more importantly, 85 proteins with increased abundance (43.14%; *P*=0.043) (Table S7) were found to be potentially targeted by at least one of the three autophagy-related proteins (P62, LC3 and ATG16L1). This indicates that the proteins with increased levels in autophagy-impaired Paneth cell organoids are targeted for degradation by selective autophagy in normal organoids, in which autophagy is functional and not compromised. Overlap analysis of the altered proteins individually targeted by p62 [upregulated in *Atg16l1*-deficient organoids as expected (Ichimura et al., 2008)], LC3 or ATG16L1 indicates that only a small proportion (19%) of the altered proteins potentially targeted by autophagy are targeted by more than one of the three autophagy proteins (Fig. 4, Table S8). This suggests that the autophagy machinery potentially mounts a coordinated effort to specifically target certain groups of proteins.

**Table 1.**
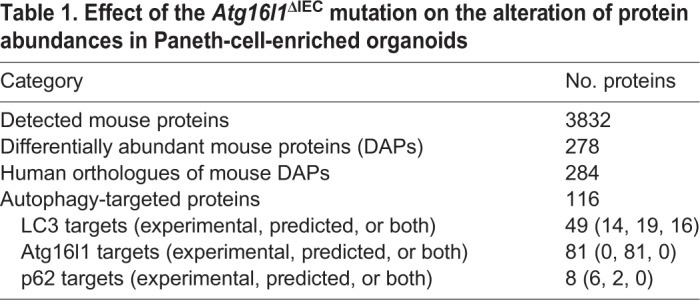
**Effect of the *Atg16l1*^ΔIEC^ mutation on the alteration of protein abundances in Paneth-cell-enriched organoids**

### Identification of Paneth cell functional processes affected by autophagy impairment

**Fig. 5.**
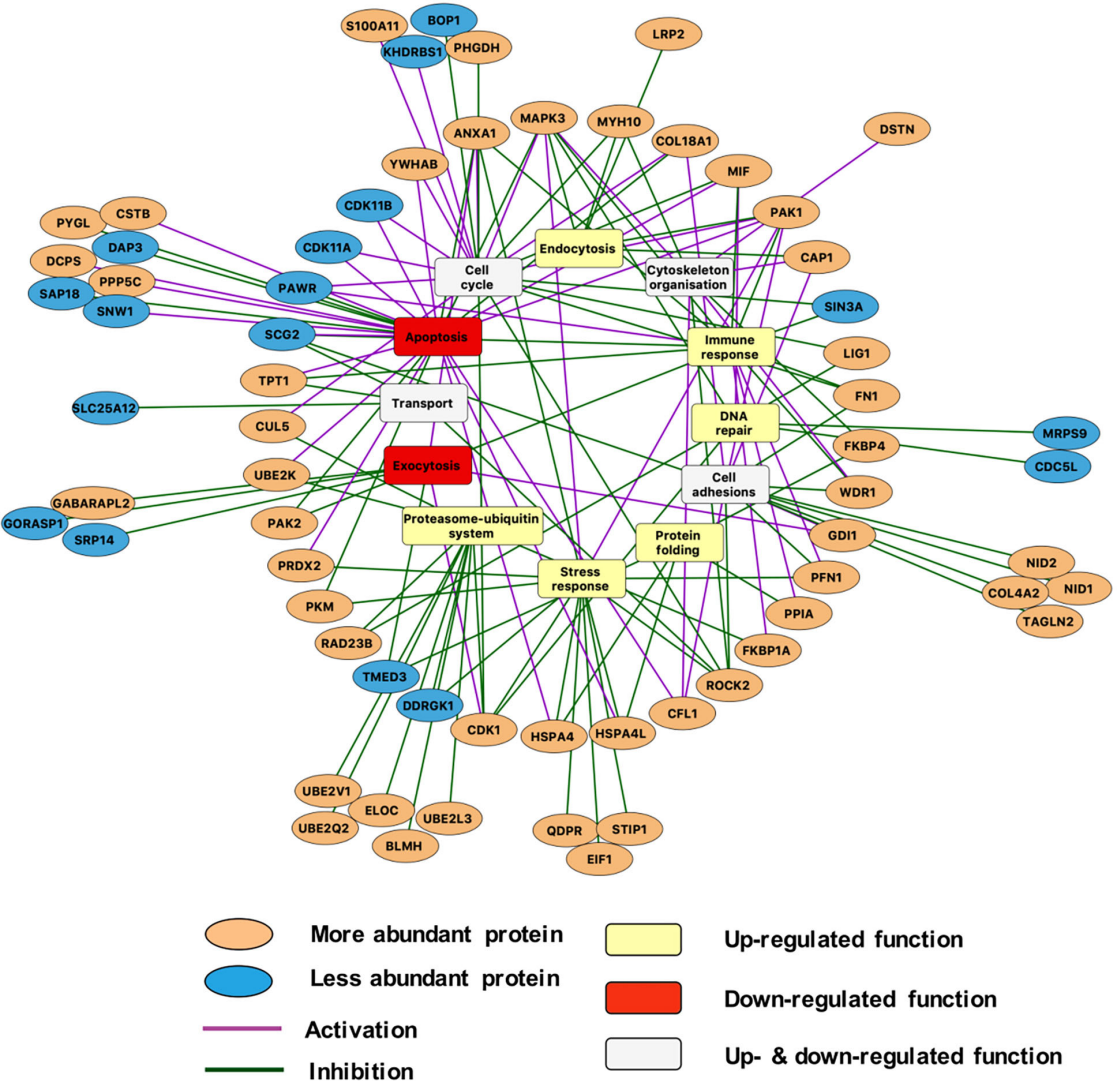
**Potential autophagy dependency of the altered functional processes inferred from the proteomic profile of the *Atg16l11*^ΔIEC^ Paneth cell organoids using our integrated approach.** The autophagy dependency of the proteins with altered abundances (orange ellipsoids for proteins with increased abundance; blue ellipsoids for proteins with decreased abundance) are represented, highlighting the effect of proteins in the processes (purple line for activation and green line for inhibition) as well. The aggregated trends of the altered functional processes as determined by the integrative approach (see Materials and Methods section) are indicated (yellow rectangles for upregulated functional processes; red rectangles for functional processes; white rectangles for functional processes that are both up- and downregulated). Proteins outside the circle are grouped to the process that they are involved in to increase the clarity of the figure. The figure was created using Cytoscape.

**Fig. 6.**
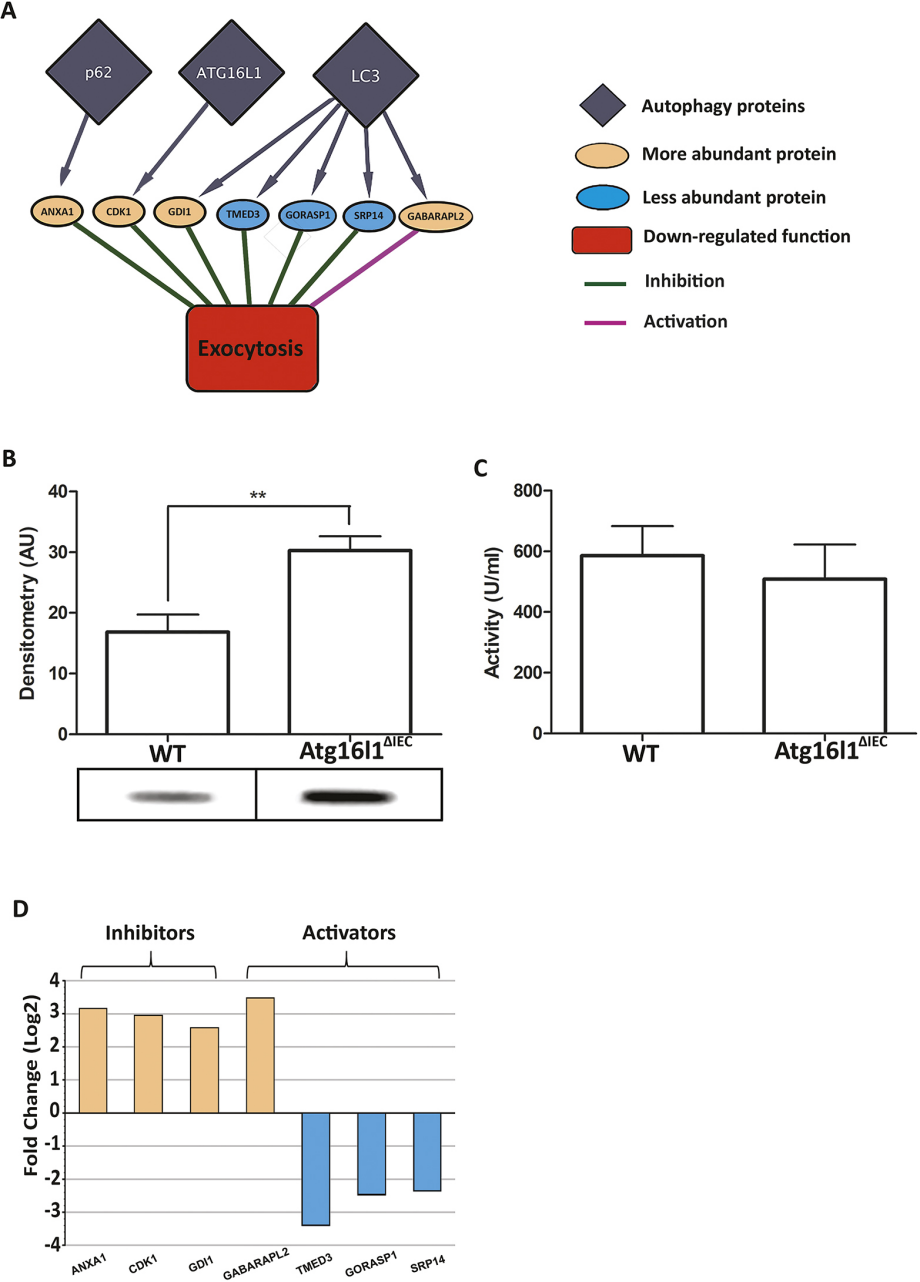
**Atg16l11 deficiency in Paneth cell organoids and its impact on exocytotic proteins (*n*=3).** (A) Proteins belonging to the functional category of exocytosis with lower abundance, reflecting the impact of autophagy impairment on Paneth cell functions such as granule processing and release through exocytosis. (B) Western blot analysis for Paneth-cell-derived lysozyme on cellular extracts from WT or *Atg16l11*^▵IEC^ organoids expressing a similar level of CD24 (Fig. 2B). **P≤0.01. (C) Lysozyme activity measured in culture medium of 2D WT and *Atg16l11*^▵IEC^ organoids as reporter of Paneth cell exocytosis. (D) Activators or inhibitors of exocytosis-related proteins found to be differentially abundant upon autophagy impairment. Blue and orange bars correspond to proteins with decreased and increased abundances, respectively. Overall, these changes support the observed reduction of exocytosis.

To determine which cellular functions could be affected due to the altered protein abundances upon autophagy impairment, we analysed the protein functions using manual curation of experimental evidence and Gene Ontology Biological Process terms (Tables S9 and S7). We identified altered functional processes, such as apoptosis, exocytosis, DNA repair etc., that could be dependent on autophagy-mediated protein degradation (Figs 5,6; Figs S1,S2; Table S10).

Since post-translational regulators can elicit positive and negative effects on functional processes, we integrated an extensive literature curation evaluating the effect of each differentially abundant protein on associated functional processes (Table S11). For each functional process, we separately calculated an aggregated trend (see Materials and Methods for details) to determine how the altered protein levels and the identified effect result in up- or downregulation of a process (Table 2). Figure 5 outlines the potential autophagy-dependent functional categories that were altered and their aggregated trends. Overall, 66 differentially abundant proteins were described by functions. Interestingly, based on the aggregated trends, we observed that 14 of the 16 altered functional processes were either uniquely upregulated or bi-directionally modulated (both up- and downregulated), while only two functional processes were uniquely downregulated (Table 2). This suggests that the overall consequence of autophagy impairment in Paneth cells is predominantly characterised by the (over)activation of various functions. These include processes such as DNA repair, endocytosis, immune response and mitochondrial organisation. Some of the upregulated functions, such as endocytosis and immune functions, have previously been directly associated with autophagy (Deretic et al., 2013; Levine et al., 2011; Tooze et al., 2014). The two uniquely downregulated functional processes, apoptosis and exocytosis, have also been associated with autophagy (Brooks et al., 2012; El-Khattouti et al., 2013; Tooze et al., 2014). We observed, for example, that 19/25 (76%) of apoptosis-related proteins that are more abundant when autophagy is impaired have an inhibitory impact on apoptosis, probably resulting in overall downregulated apoptosis (Fig. S1). Similarly, 5/7 (>71%) of DNA-repair-related proteins that are more abundant upon autophagy alteration have an activating impact on DNA repair (Fig. S2). Thus, taken as an extension to previous findings, our results show that autophagy-mediated protein degradation can regulate key Paneth cell functions, such as exocytosis, and potentially affect the activity of apoptosis regulators.

**Table 2.**
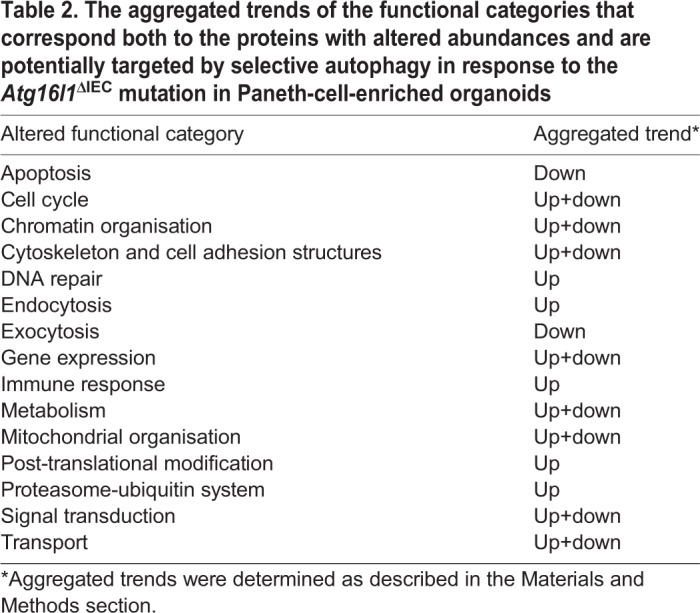
**The aggregated trends of the functional categories that correspond both to the proteins with altered abundances and are potentially targeted by selective autophagy in response to the *Atg16l1*^ΔIEC^ mutation in Paneth-cell-enriched organoids**

### Validation of autophagy effect on protein degradation using transcriptomic data analysis

Change in abundance of proteins can be a consequence of altered gene expression, protein production and protein turnover. To validate that the observed difference due to autophagy impairment is mediated through protein turnover and not gene expression changes, we measured the transcriptomic profiles of Paneth-cell-enriched organoids derived from mice lacking or not Atg16l1 in their intestinal epithelium. Comparing the FC value of genes and proteins that were present in both proteomic and transcriptomic profiles can help to understand whether changes in protein abundance depend on gene expression or on protein turnover. The genes coding for the 66 differentially abundant proteins that are predicted autophagy targets (LC3, p62 or ATG16L1 binding partners) were therefore analysed from transcriptomic data generated on additional organoid samples (Table S12). As a result, we found that 44 proteins, from the 66 differentially abundant candidates, were also differentially transcribed (Table S12). However, most of them (40/44 proteins) showed noticeable differences at both transcriptional and protein levels using the cut-off value defined in the Materials and Methods section: in every case, the FC value for the transcriptomic data was much lower than the change in abundance of the protein expressed from the gene. Therefore, we assume that changes in protein abundance level happen due to impaired autophagy-mediated protein degradation.

### Impact of autophagy impairment on exocytosis in Paneth cells

As secretory cells, Paneth cells are reliant on high levels of protein biosynthesis and secretion, the latter being strongly reliant on functional autophagy. We assessed whether the autophagy impairment had an effect on the levels of proteins associated with exocytosis. Interestingly, our integrative analysis (using the workflow explained in Fig. 1) of the proteomic response revealed that exocytosis could be repressed in the absence of functional autophagy. Figure 6A shows the altered levels of exocytosis proteins in *Atg16l1*^▵IEC^ Paneth cell organoids as well as the autophagy-targeting proteins, which could be modulating them. This result is in agreement with the already established importance of autophagy in the exocytosis-mediated secretion of antimicrobial peptides (AMPs) (Brooks et al., 2012; Cadwell et al., 2008, 2009a,b; Gassler, 2017; Tschurtschenthaler and Adolph, 2018). We also determined experimentally that lysozyme levels detected within organoids were significantly greater when autophagy was impaired (Fig. 6B) than in WT organoids. However, levels of lysozyme secreted into the culture medium were slightly reduced in *Atg16l1*^▵IEC^ organoids, although not significantly (Fig. 6C), suggesting a defective exocytosis pathway upon impaired autophagy. Detailed analysis of our proteomic data showed that proteins targeted by LC3, Atg16l1 and p62 (Fig. 4) and involved in the inhibition of exocytosis were found to be more abundant when autophagy was impaired. The opposite effect was observed, with autophagy-targeted proteins involved in the activation of exocytosis being less abundant, upon autophagy impairment (Fig. 6D). This agrees with the negative alteration of exocytosis of AMPs that we observed in our validation assays (Fig. 6B,C).

The exocytotic pathways facilitating the secretion of proteins are mediated either by general mechanisms involving the ER and the Golgi apparatus (Barlowe and Miller, 2013) in an autophagy-independent manner, or, in the case of antimicrobial proteins, through the recently discovered LC3- and autophagy-dependent secretory process (Bel et al., 2017; Ponpuak et al., 2015). So far, it was not clear whether these two exocytotic pathways are co- or independently regulated, nor whether they share some of the proteins involved and target overlapping proteins. Here, we revealed that proteins with exocytosis functions having higher abundance levels upon impaired autophagy could be potential autophagy targets; these include SRP14 (signal recognition particle 14 kDa protein), GORASP1 (Golgi reassembly-stacking protein 1) and TMED3 (transmembrane emp24 domain-containing protein 3; Fig. 6D; Table S13). This unexpected result raises the question of whether to revisit the autophagy relatedness of the ER/Golgi-specific pathway, which shunts lysozyme into secretory granules that are involved in exocytosis. Based on these observations, it is plausible that autophagy could have a direct effect on processes that are thought to be autophagy independent.

## DISCUSSION

Using a multidisciplinary combinatorial approach generating integrating interaction networks from proteomic data from murine Paneth-cell-enriched organoids, interaction networks and validatory experiments, we revealed Paneth cell functional processes that are dependent on autophagy. Atg16l1 has been described as a pivotal autophagy protein in the last decade and it was shown that dysfunctional Atg16l1 leads to impaired formation of autophagosomes and poor degradation of long-lived proteins (Kaser and Blumberg, 2014; Saitoh et al., 2008). Our study focused on the role of autophagy in Paneth cell homeostasis, in particular on the consequences of impaired autophagy on Paneth cell functions. We used Paneth-cell-enriched organoids derived from mice lacking the *Atg16l1* gene specifically in intestinal epithelial cells. Although this model may present the inconvenience of not being entirely composed of Paneth cells, and sorting Paneth cells from three-dimensional (3D) organoids would have been an equally valid option, it would have been technically more challenging in view of generating a sufficient amount of material for proteomic analysis. Furthermore, it was recently shown that *in vitro* cultured organoids enriched for a specific cell type such as Paneth cells exhibit features that better recapitulate functions of *in vivo* Paneth cells than normally differentiated organoids (Mead et al., 2018). Using a *lyz-Cre* mouse model in future studies in combination with single-cell transcriptome profiling will also confirm the impact of autophagy impairment that we measured on Paneth cells.

Atg16l1 is an important component of the autophagy machinery whose human orthologue was previously associated with digestive pathologies such as CD. We determined quantitative proteomic profiles of Paneth-cell-enriched organoids with functional or impaired autophagy. We developed and applied a computational systems-biology approach based on the analysis of proteomic profiles and integrated multiple types of already existing but so-far unconnected disparate information (protein-protein interaction networks, information about proteins known to be targeted by autophagy and functional information about proteins displaying differential abundance when autophagy was impaired). Integration of these data with the interaction networks of selective autophagy receptors and adaptors, such as p62, LC3 and ATG16L1, helped relate the degradation of the altered proteins to their regulation by autophagy. Furthermore, by incorporating known functions and biological processes attributed to the affected proteins, we identified various cellular processes that could be dependent on functional autophagy.

As recently reported for stem-cell-enriched organoids, our study emphasises the robustness of systems-level approaches to fully capture the impact of major impaired cellular processes – in our case, autophagy – on homeostatic cellular functions (Lindeboom et al., 2018). The computational pipeline presented in the current study enabled the building of regulatory networks of proteins displaying differential abundance upon autophagy impairment. To overcome the lack of mouse protein-protein interaction information involving the autophagy receptor and adaptor proteins, as well as to exploit the corresponding information already available in human datasets, we used the human orthologues of the mouse proteins with altered abundances in mouse-derived *Atg16l1*^▵IEC^ organoids. Although the cross-species extrapolation could be a source of uncertainties and possible missing information, the identified processes and their direction of modulation concur to a certain extent with already existing knowledge about the effects of autophagy impairment. Other notable limitations of our study include the inability of the proteomic measurements to distinguish between the two isoforms of LC3 – LC3I and LC3II – thereby hindering interpretations about the role of the isoforms.

Strikingly, our analysis revealed that, when autophagy is impaired upon lack of *Atg16l1*, nearly 300 proteins display increased or decreased abundance, encompassing at least 18 functional processes (Fig. 3). Transcriptomic analysis was carried out on Paneth-cell-enriched organoids to identify the level of modulation of affected cellular processes. Most of the potential autophagy-targeted proteins exhibited massive abundance discrepancies upon autophagy impairment but relatively small differential expression at the transcriptional level, confirming the strikingly stronger effect that autophagy has on protein-level regulation rather than on transcriptional regulation. Among the altered proteins, several had previously been associated with pathologies affecting Paneth cells, such as ANXA1 and FGA, which were previously reported to be altered in inflamed mucosal tissue or epithelial cells from CD patients (Barceló-Batllori et al., 2002; Iskandar and Ciorba, 2012; Meuwis et al., 2007). We observed that, when autophagy is impaired in Paneth cells, most of the differentially abundant proteins are present in greater abundance than in normal Paneth cells, thus suggesting that degradation through autophagy plays a key role in maintaining the intracellular concentrations of these proteins.

The developed computational pipeline enhances our understanding about the underlying mechanisms involved in autophagy-mediated degradation by integrating multiple levels of information, such as protein-protein interactions, autophagy-mediated selective protein degradation, inhibitory/stimulatory relationships between the altered proteins and functional processes. Notably, to reduce the impact of linear assumptions in interpreting the impacts of proteomic changes on functional processes, we determined the aggregated trends for the functional processes by incorporating the direction of protein-level alterations and the stimulatory/inhibitory relationships between the altered proteins and the functions. Furthermore, by bringing together different levels of information, our approach helps explain the mechanistic underpinnings between the processes corresponding to the proteins with altered abundances and autophagy. Capturing such process-level dependencies on cellular autophagy and their modulation would be difficult by using singular levels of information in isolation. For example, Zhang et al. measured the proteome-level changes in primary human fibroblasts that were impaired in autophagy as a means to explain the purported dependency of protein degradation on macroautophagy (Zhang et al., 2016). Patella et al. identified proteomic alterations under conditions of autophagy blockage in endothelial cells to explain the potential role of autophagy in maintaining endothelial permeability (Patella et al., 2016). Similarly, various other studies have profiled the global proteomic changes in response to artificial impairment of autophagy by knocking out critical autophagy genes (Avin-Wittenberg et al., 2015; Mathew et al., 2014). Studies combining different -omic readouts have also been performed in various contexts to understand the role of autophagy in various processes and phenotypes (Chen et al., 2017; Kramer et al., 2017; Masclaux-Daubresse et al., 2014; Stingele et al., 2012). However, the aforementioned studies do not provide an explanation as to how the proteome-level alterations are indeed dependent on autophagy from a mechanistic point of view. In this study, using networks and integration of heterogeneous datasets, we provide information on new mechanisms by which several cellular processes, such as exocytosis, DNA repair and apoptosis, are dependent on autophagy.

Upon microbial invasion or inflammation-mediated cellular damage, cells respond by activating apoptotic cell death. In general, autophagy and apoptosis are negatively correlated under most homeostatic conditions (Mariño et al., 2014), although altered cellular settings can drive autophagy to lead to programmed cell death. The interactions between autophagy and apoptosis are highly complex (Gump and Thorburn, 2011; Mariño et al., 2014). Interestingly, our observations showed a positive correlation between autophagy and apoptosis (with apoptosis being inhibited in the autophagy-impaired organoids; Fig. S1). When autophagy is impaired, the observed downregulation of apoptosis could prevent the perturbed Paneth cells from sacrificing themselves, which would then be compensated for by outcomes such as upregulation of DNA-damage-repair functions as suggested previously (Basu and Krishnamurthy, 2010; Nowsheen and Yang, 2012; Fig. S2). However, further experiments are needed to confirm the assumption about the role of DNA repair and apoptosis in Paneth cells and how the disruption of these processes could contribute to the pathogenesis of impaired autophagy-associated diseases.

ATG16L1 is known to be required not only for the normal functioning of autophagy, but it also has physiological relevance. The intestinal epithelium in patients with inflammatory digestive disorders is characterised by a prolonged period of stress as a result of chronic inflammation and malfunction of antimicrobial innate defences. This is reflected in particular by the alteration of exocytosis of antimicrobials as well as the manipulation of the genetic/epigenetic machinery and organelles by invading pathogens and various other causative agents (Fofanova et al., 2016; Sartor, 2006). In this study, the downregulation of exocytosis that we observed in autophagy-impaired organoids was illustrated by lysozyme accumulation within organoid cells and the consequential alteration of lysozyme secretion into the extracellular milieu. These results support our computational analysis and concur with the previously reported autophagy dependency of exocytosis (Brooks et al., 2012; Cadwell et al., 2008, 2009a,b; Gassler, 2017; Tschurtschenthaler and Adolph, 2018) (Figs 4 and 6). Impaired autophagy can therefore have dramatic consequences on innate defence mechanisms against microbial invasion of the gut epithelium by deregulating the protein degradation of key exocytotic proteins.

The Paneth-cell-enriched organoids that we derived from the *Atg16l1*^▵IEC^ mouse model could be perceived as a biased representation of the role that autophagy impairment has in inflammatory diseases (infectious or chronic), as it does not consider other intrinsic factors, such as mutations in non-autophagy-related genes, that have been shown to contribute to those pathologies (e.g. proteins such as PARP-2, IFI35, S100A12, CRP and S100A8 have been previously shown to contribute to IBD pathogenesis) (Cheluvappa et al., 2014; de Jong et al., 2006; Jijon et al., 2000; Leach and Day, 2006; Vermeire et al., 2006). These biomarkers are indicative of an inflammatory state; some of them are mostly detected in the serum, but indicators detected in faecal samples predict more accurately the state of digestive inflammatory state. For example, inhibition of PARP dampens inflammation associated with colitis (Jijon et al., 2000), while elevated levels of CRP and S100A8, for example, have been associated with inflammatory pathologies (Wang et al., 2018). Yet, in our study, these proteins were not found to be differentially abundant when autophagy was impaired, or to fluctuate in the opposite direction. These discrepancies could reflect the differences in the sample type as these studies did not focus on Paneth cells only. Complementary experiments and predictions would nonetheless help to highlight some of the aspects of the molecular regulatory mechanisms that contribute to the pathogenesis of digestive diseases upon alteration of autophagy.

Our integrative analysis not only captured already known phenomena, namely the autophagy dependency of exocytotic functions associated with granule release, but also highlighted that the degradation regulation of many differentially abundant proteins, including those of exocytosis proteins occurring in an autophagy-dependent manner. The presented study therefore extended the list of proteins for which the degradation rate was already known to be regulated by autophagy. More interestingly, our analysis revealed additional cellular processes that could mediate the effects of autophagy impairment on Paneth cell functions. Taken together and using a mouse model where autophagy is impaired, and organoids to capture Paneth cells, we identified various cellular processes that are dependent on autophagy and whose failure could further contribute to the pathogenesis of major digestive pathologies.

## MATERIALS AND METHODS

### Animal handling

C57/BL6 mice of both sexes were used for organoid generation. All animals were maintained in accordance with the Animals (Scientific Procedures) Act 1986 (ASPA).

### Generation of *Atg16l1*^flox/− Vil-Cre^ (*Atg16l1*^ΔIEC^) mice

Mice were generated using a Cre/*loxP* system. Briefly, *loxP* sites were inserted either side of *Atg16l1* exon 2, creating *Atg16l1*^flox/+^ mice. Crossing these mice with phosphoglycerate kinase (*PGK*)-Cre mice, expressing Cre recombinase under the *PGK* promoter, which is in all cell types, led to the excision of the floxed exon 2 in one allele by Cre recombinase. This in turn introduced a stop codon, generating *Atg16l1*^+/−^ mice. Cell-type-specific *Atg16l1* deletion was induced using a villin promoter to drive expression of Cre recombinase only in villin-expressing cells. This was achieved by crossing *Atg16l1*^flox/flox^ mice with *Atg16l1*^+/−^^V^^il-Cre^ mice to produce *Atg16l1*^flox/− Vil-Cre^ mice deficient in *Atg16l1* in intestinal epithelial cells. *Atg16l1^flox^*^/+^ mice were used as wild-type controls (WT). Transgenic mice were genotyped using end-point PCR and gel electrophoresis. *Atg16l1* alleles were firstly designated as WT (+), floxed (Fl) or knockout (−) and the presence or absence of the Cre recombinase gene under the control of the villin promoter subsequently designated positive (Vil-Cre) or negative. Combining the PCR results identified the *Atg16l1*^flox/+^ (WT) or *Atg16l1*^flox/− Vil-Cre^ (*Atg16l1*^▵IEC^ KO) mice. All primers used to validate the organoid models are listed in Table S1.

### Small intestinal organoid cultures for both proteomic and transcriptomic profiling

Murine small-intestinal organoids were generated as described previously (Sato et al., 2013). Briefly, the entire small intestine was opened longitudinally, washed in cold PBS, then cut into ∼5 mm pieces. The intestinal fragments were incubated in 30 mM EDTA/PBS for 5 min, transferred to PBS for shaking, then returned to EDTA for 5 min. This process was repeated until five fractions were generated. The PBS supernatant fractions were inspected for released crypts. The crypt suspensions were passed through a 70 μm filter to remove any larger villus-containing fragments, then centrifuged at 300 ***g*** for 5 min. For 3D organoid cultures, pellets were resuspended in 200 μl Phenol-Red-free Matrigel (Corning), seeded in small domes into 24-well plates and incubated at 37°C for 20 min to allow Matrigel to polymerise. Organoid medium [Advanced DMEM/F12 (Life Technologies)] containing growth factors including EGF (50 ng/ml, Life Technologies), noggin (100 ng/ml, PeproTech) and R-spondin 1 (500 ng/ml, R&D Systems) was then overlaid. For 2D organoid monolayers used for the lysozyme secretion validation, pellets were resuspended in organoid media and overlaid onto coverslips coated with Phenol-Red-free Matrigel (Corning). For the quantitative proteomics analysis, the Paneth cell population in 3D WT and *Atg16l1*^ΔIEC^ organoids were enriched by addition of 3 μM CHIR99021 (Tocris) and 10 μM DAPT (Tocris) to the organoid culture media on day 2, 5 and 7 post-crypt-isolation according to previously published and well-established protocols (Nakanishi et al., 2016; Yin et al., 2014).

For both proteomic and transcriptomic profiling, replicate organoids were generated from three individual animals for each genotype and each enrichment condition tested, generating three biological replicates for each sample group. Two additional biological replicates of both WT normally differentiated and Paneth-cell-enriched organoids were also generated, profiled by proteomics and used as controls to validate Paneth cell enrichment. One replicate was subsequently removed from the WT Paneth-cell-enriched transcriptomics dataset as initial analysis showed it as an outlier.

### RT-PCR, qPCR and immunoblotting

To confirm that WT and *Atg16l1*^ΔIEC^ organoids retained the intestinal phenotype and expressed the intestinal epithelial-cell-type markers, gene expression was analysed by RT-PCR. On day 8 post-crypt-isolation, normally differentiated organoid pellets were lysed in 500 µl TRIzol (Life Technologies). RNA was extracted by chloroform extraction followed by precipitation in isopropanol and ethanol. Total RNA (200 ng) isolated from organoids was reversed transcribed to generate single-stranded cDNA. Gene-specific primers for *Lgr5* (stem cells), villin (epithelial cells), chromogranin A (enteroendocrine cells), mucin 2 (goblet cells), *CD24* (Paneth cells) and β-actin were used for linear amplification of cDNAs using a limited number of cycles. β-actin was used as a housekeeping gene for expression analysis. Primers and PCR cycle numbers are listed in Table S1. To confirm the absence of the Atg16l1 protein, impairment in autophagy and lysozyme quantification, specific protein expression was analysed by immunoblotting and western blot analysis. On day 8 post-crypt-isolation, normally differentiated or Paneth-enriched organoid pellets were lysed in m-PER lysis buffer (Thermo Fisher Scientific) containing protease inhibitor cocktail (Roche). A total of 3-30 µg protein per well was separated using a NuPAGE precast 4-12% polyacrylamide gel system (Thermo Fisher Scientific). Immunoblotting was carried out using an X-Cell II blot module (Thermo Fisher Scientific) onto polyvinylidene (PVDF) membranes (Thermo Fisher Scientific). Membranes were probed for ATG16L1 (Abgent #AP1817b), LC3II (Sigma-Aldrich #L7543), β-actin [Sigma-Aldrich #A1978 (clone AC015)] and lysozyme (Dako #A0099 EC3.2.1.17), and protein bands visualized using Odyssey infrared imaging system (Li-Cor) at 700 nm and 800 nm. Densitometry of lysozyme bands was performed using the FIJI/ImageJ package and expressed as arbitrary units (AU) from at least three biological replicates for each group.

### Protein sample preparation for proteomics

On day 8 post-crypt-isolation, Paneth-cell-enriched 3D organoids were extracted from Matrigel using Cell Recovery Solution (BD Bioscience), washed in PBS and centrifuged at 300 ***g*** for 5 min. Organoid pellets were lysed by sonication in 1% (w/v) sodium deoxycholate (SDC) in 50 mM ammonium bicarbonate. Samples were heated at 80°C for 15 min before centrifugation at 12,000 ***g*** to pellet debris. The supernatant was retained, and proteins reduced with 3 mM DTT (Sigma-Aldrich) at 60°C for 10 min, cooled, then alkylated with 9 mM iodoacetamide (Sigma-Aldrich) at room temperature for 30 min in the dark; all steps were performed with intermittent vortex-mixing. Proteomic-grade trypsin (Sigma-Aldrich) was added at a protein:trypsin ratio of 50:1 and incubated at 37°C overnight. SDC was removed by adding trifluoroacetic acid (TFA) to a final concentration of 0.5% (v/v). Peptide samples were centrifuged at 12,000 ***g*** for 30 min to remove precipitated SDC.

### NanoLC MS electrospray ionization (ESI) MS/MS analysis

Peptides were analysed by online nanoflow LC using the Ultimate 3000 nano system (Dionex/Thermo Fisher Scientific) coupled to a Q-Exactive HF mass spectrometer (Thermo Fisher Scientific) essentially as described in Dong et al. (2017). Peptides were separated by an Easy-Spray PepMap^®^ RSLC analytical column (50 cm×75 μm inner diameter, C18, 2 μm, 100Å) fused to a silica nano-electrospray emitter (Dionex). Column temperature was kept at a constant 35°C. Chromatography buffers consisted of 0.1% formic acid (buffer A) and 80% acetonitrile in 0.1% formic acid (buffer B). The peptides were separated by a linear gradient of 3.8-50% buffer B over 90 min at a flow rate of 300 nl/min. The Q-Exactive HF was operated in data-dependent mode with survey scans acquired at a resolution of 60,000. Up to the top ten most-abundant isotope patterns with charge states +2 to +5 from the survey scan were selected with an isolation window of 2.0 Th and fragmented by higher-energy collisional dissociation with normalised collision energies of 30. The maximum ion injection times for the survey scan and the tandem mass spectrometry (MS/MS) scans were 100 and 45 ms, respectively, and the ion target value was set to 3E6 for survey scans and 1E5 for the MS/MS scans. MS/MS events were acquired at a resolution of 30,000. Repetitive sequencing of peptides was minimised through dynamic exclusion of the sequenced peptides for 20 s.

### Protein identification and quantification

Spectral data was imported into Progenesis QI (version 4.1, Nonlinear Dynamics). Runs were time aligned using default settings and using an auto-selected run as reference. Peaks were picked by the software using default settings and filtered to include only peaks with a charge state between +2 and +7. Spectral data were converted into .mgf files with Progenesis QI and exported for peptide identification using the Mascot (version 2.3.02, Matrix Science) search engine. MS/MS data were searched against a database including translated open reading frames (ORFs) from the *Mus musculus* genome [Uniprot reference proteome (reviewed), |UP000000589, February 2017] and a contaminant database (cRAP, GPMDB, 2012) (combined 17,010 sequences; 9,549,678 residues). Precursor mass tolerance was set to 10 ppm and fragment mass tolerance was set as 0.05 Da. Two missed tryptic cleavages were permitted. Carbamidomethylation (cysteine) was set as a fixed modification and oxidation (methionine) set as a variable modification. Mascot search results were further validated using the machine-learning algorithm Percolator embedded within Mascot. The Mascot decoy database function was utilised and the false discovery rate (FDR) was <1%, while individual percolator ion scores >13 indicated identity or extensive homology (*P*<0.05). Mascot search results were imported into Progenesis QI as XML files. Peptide intensities were normalised against the reference run by Progenesis QI and these intensities were used to highlight relative differences in protein expression between sample groups. Only proteins with two or more identified peptides were included in the dataset. Statistical analysis (one-factor ANOVA) of the data was performed using Progenesis QI to identify proteins with significantly altered abundances (*P*<0.05, absolute relative FC ≥2, number of unique peptides ≥2). The proteomic dataset has been submitted to PRIDE (accession id: PXD010940).

### RNA sample preparation for transcriptomics

For transcriptomic profiling, Paneth-cell-enriched organoids were extracted from Matrigel on day 8, recovered in Cell Recovery Solution (BD Bioscience) and washed in PBS. RNA extraction was performed using the Exiqon tissue kit according to the manufacturer's protocol. Stranded RNA-seq libraries were constructed using the NEXTflex™ Rapid Directional RNA-Seq Kit (PN: 5138-07) using the poly-A pull-down beads from Illumina TruSeq RNA v2 library construction kit (PN: RS-122-2001) with the NEXTflex™ DNA Barcodes – 48 (PN: 514104) diluted to 6 µM. RNA quality control (QC) was carried out using the Qubit DNA kit (Life Technologies Q32854 and Q32852) and PerkinElmer GX RNA assay (PN:CLS960010) prior to library construction. In more detail, mRNAs were extracted with a poly-A pulldown using biotin beads, fragmented, and first-strand cDNA was synthesised. This process reverse transcribes the cleaved RNA fragments primed with random hexamers into first-strand cDNA using reverse transcriptase and random primers. The second-strand synthesis process removes the RNA template and synthesises a replacement strand to generate double-stranded cDNA. Directionality is retained by adding dUTP during the second-strand synthesis step and subsequent cleavage of the uridine-containing strand using Uracil DNA Glycosylase. The ends of the samples were repaired using the 3′-to-5′ exonuclease activity to remove the 3′ overhangs and the polymerase activity to fill in the 5′ overhangs creating blunt ends. A single ‘A’ nucleotide was added to the 3′ ends of the blunt fragments to prevent them from ligating to one another during the adapter ligation reaction. A corresponding single ‘T’ nucleotide on the 3′ end of the adapter provided a complementary overhang for ligating the adapter to the fragment. This strategy ensured a low rate of chimera formation. The ligation of a number of indexing adapters to the ends of the DNA fragments prepared them for hybridisation onto a flow cell. The ligated products were subjected to a bead-based size selection using Beckman Coulter XP beads (PN: A63880). As well as performing a size selection, this process removed the majority of unligated adapters. Prior to hybridisation to the flow cell, the samples underwent PCR to enrich for DNA fragments with adapter molecules on both ends and to amplify the amount of DNA in the library. The strand that was sequenced is the cDNA strand. The insert size of the libraries was verified by running an aliquot of the DNA library on a PerkinElmer GX using the High Sensitivity DNA chip (PerkinElmer CLS760672) and the concentration was determined by using a High Sensitivity Qubit assay and RT-PCR. Libraries were then equimolar pooled and checked by RT-PCR to ensure that they had the necessary sequencing adapters ligated.

### Sequencing the stranded RNA libraries

The constructed stranded RNA libraries were normalised and equimolar pooled, and the final pool was quantified using a KAPA Library Quant Kit and found to be 2 nM for the WT samples and 9.8 nM for the *Atg16l1*^▵IEC^ samples. A total of 9.5 µl (WT) and 2.04 µl (*Atg16l1*^ΔIEC^) of the pool was combined with 0.5 µl 2N NaOH to make a 2 nM dilution. This was incubated for 5 min at room temperature to denature the libraries before 990 µl of HT1 was added to make a 20 pM dilution. A total of 60 µl of the 20 pM dilution was combined with 60 µl of HT1 plus a 1% PhiX spike in Illumina FC-110-3001. For each lane the pool was run in to make the final running concentration of 10 pM. The flow cell was clustered using HiSeq PE Cluster Kit v3 (Illumina PE-401-3001) for the WT samples and HiSeq PE Cluster Kit v4 (Illumina GD-401-4001) for the *Atg16l1*^ΔIEC^ samples. The Illumina PE_HiSeq_Cluster_Kit_V3_cBot_recipe_V8.0 (WT) and PE_HiSeq_Cluster_Kit_V4_cBot_recipe_V9.0 (*Atg16l1*^ΔIEC^) methods were used on the Illumina cBot. Following the clustering procedure, the flow cell was loaded onto the Illumina HiSeq2000 (WT) or HiSeq2500 (*Atg16l1*^ΔIEC^) instrument following the manufacturer's instructions with a 101-cycle paired read and a 7-cycle index read (WT) or a 126-cycle paired end read and a 12 bp/6 bp dual-index read (*Atg16l1*^ΔIEC^). For WT samples, the sequencing chemistry used was HiSeq SBS Kit v3 (Illumina FC-401-3001) with HiSeq Control Software 2.2.68 and RTA 1.18.66.3. For *Atg16l1*^ΔIEC^ samples, the sequencing chemistry used was HiSeq SBS Kit v4 (Illumina FC-401-4003) with HiSeq Control Software 2.2.58 and RTA 1.18.64. Reads in bcl format were demultiplexed based on the 6 bp Illumina index by CASAVA 1.8, allowing for a one-base-pair mismatch per library, and converted to FASTQ format by bcl2fastq.

### Mapping and identification of differentially expressed transcripts

The quality of stranded reads was assessed by FastQC software (version 0.11.4). Gene and transcript abundances were estimated with kallisto (version 0.44.0) (Bray et al., 2016). The Sleuth R library was used to perform differential gene expression (0.30.0) (Pimentel et al., 2017). mRNAs and lncRNAs with an absolute log_2_ FC of 1 and *q*-value ≤0.05 were considered to be differentially expressed.

### Lysozyme activity assay

Lysozyme activity associated with Paneth cell antimicrobial defence was measured in 2D organoid culture medium using the EnzChekTM Lysozyme Assay Kit, according to the manufacturer's instructions (Thermo Fisher Scientific). Briefly, 2D organoids were cultured from WT and *Atg16l1*^ΔIEC^ mice as described in the third Materials and Methods section. Following a 20 h post-seeding incubation, the organoid culture medium was collected. Remaining cellular debris was removed by centrifugation at 600 ***g*** for 5 min and filtration on 0.20 µm PES filters. FITC-fluorescence, proportional to the lysozyme activity released by Paneth cells into the medium was measured on a Fluostar Optima Fluorometer (BMG Labtech) and corrected for background fluorescence. Lysozyme activity expressed as U/ml was determined from standard curves on at least three biological replicates for each group.

### Cross-validation of transcriptomic and proteomic profiles

To compare the quantity of gene expression and protein abundance, Ensembl gene IDs were converted to UniProt IDs to unify the identities using the ID mapping tool of UniProt. We defined a cut-off value: if the difference between the log_2_-based FC values (regarding the protein abundance and gene expression) is higher than 0.7, hypothetically the change in protein abundance level could happen due to the malfunction of autophagy.

### Interaction resources and computational methods to identify proteins targeted by autophagy

In order to make the proteomic data comparable to human interaction networks, the human orthologues of the proteins with altered abundances from the Paneth cell organoids were identified using InParanoid (Sonnhammer and Östlund, 2015). To identify the autophagy-targeted protein components, the interaction partners of the three autophagy receptor and adaptor proteins, namely p62, LC3 and ATG16L1, were retrieved from the manually curated section of the Autophagy Regulatory Network (Türei et al., 2015). To enhance the coverage and improve interpretations, the interactions retrieved from experimental data were complemented with the predicted targets of p62, LC3 and ATG16L1. The putative targets of p62 and ATG16L1 were inferred using in-house custom scripts written in the Python programming language. The predictions of p62 and ATG16L1 targets were based on the standard motif search and domain-domain interaction prediction methods (Korcsmaros et al., 2013), respectively. The p62 recognition motif was retrieved from Jadhav et al. (2011). For the domain-domain interaction prediction method, the known set of interacting domain pairs were obtained from the DOMINE database (Raghavachari et al., 2008). DOMINE captures information about interacting domain pairs from experiments, structural studies and predictions. Domain annotations for all proteins were retrieved from UniProt (The UniProt Consortium, 2017). The targets of LC3 were downloaded from the iLIR database (Jacomin et al., 2016).

### Functional analysis to identify affected processes

To check whether the changes are due to Paneth cell enrichment or because of impaired autophagy, we carried out a control experiment where we compared the proteomic profile of normal intestinal organoid to Paneth-cell-enriched organoids. To assess the functional importance of proteins with altered abundances targeted by autophagy within a network context, Gene Ontology Biological Process terms (Ashburner et al., 2000) derived from UniProt (The UniProt Consortium, 2017) were used. Biological Process terms not related to the intestine (action potential, development of other organs/tissues etc.) were discarded following a manual curation of the terms in order to maintain the intestinal context (Table S2). We performed an additional, extensive manual curation to determine the role of all the proteins in these biological processes as well as to define their effect (activational or inhibitory) on these processes. Based on the directionality of the interaction between p62/LC3/ATG16L1 and the proteins with altered abundances, we inferred the direction of modulation of the assigned Biological Process terms. The aggregated trends of the processes were inferred as follows: the processes were considered to be either upregulated or downregulated if more than 70% of its interactions were classified as stimulatory or inhibitory, respectively. In cases where the singular proportion is less than 70%, the functional process was considered to have dual modulation (i.e. both up- and downregulated). The networks were visualised in Cytoscape 3.5.1 (Franz et al., 2016).

## Supplementary Material

Supplementary information
