# Supplementary material for: Integrative analysis of Paneth cell proteomic and transcriptomic data from intestinal organoids reveals functional processes dependent on autophagy
Source: Dis Model Mech. 2019 Mar 18;12(3):dmm037069. doi: 10.1242/dmm.037069 (PMC6451430; doi:10.1242/dmm.037069)
Supplement: Supplementary information [file dmm-12-037069-s1.pdf]

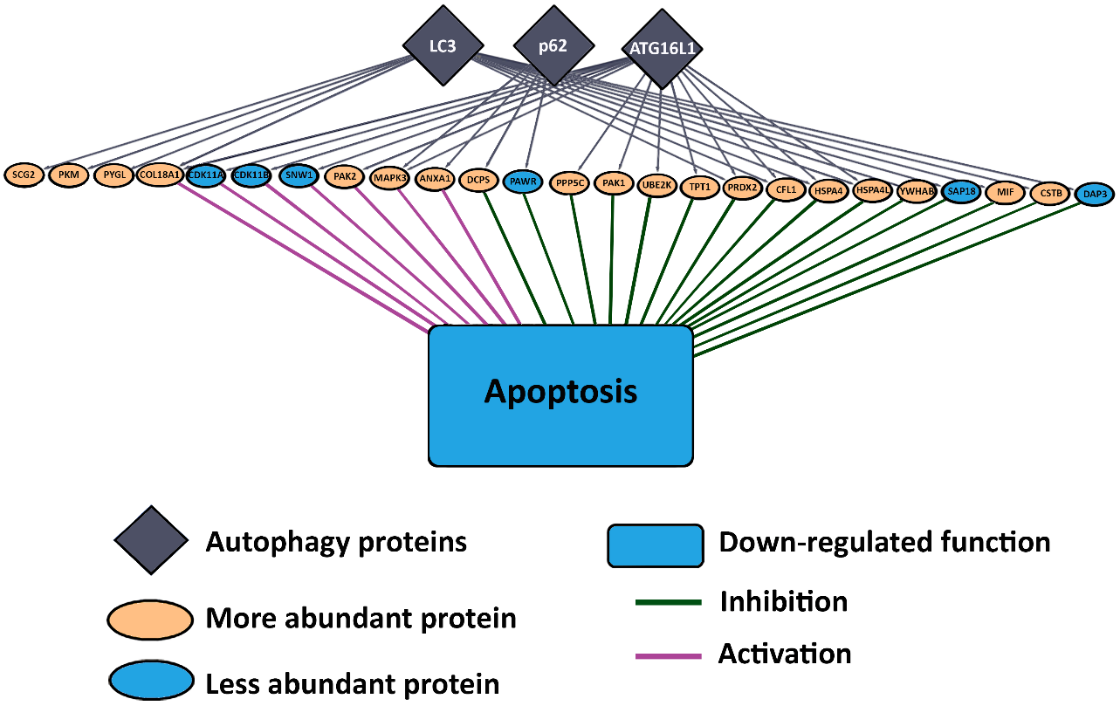

Figure S1. Alterations describing the modulation of apoptosis in response to the *Atg16L1*$^{\Delta IEC}$ mutation.

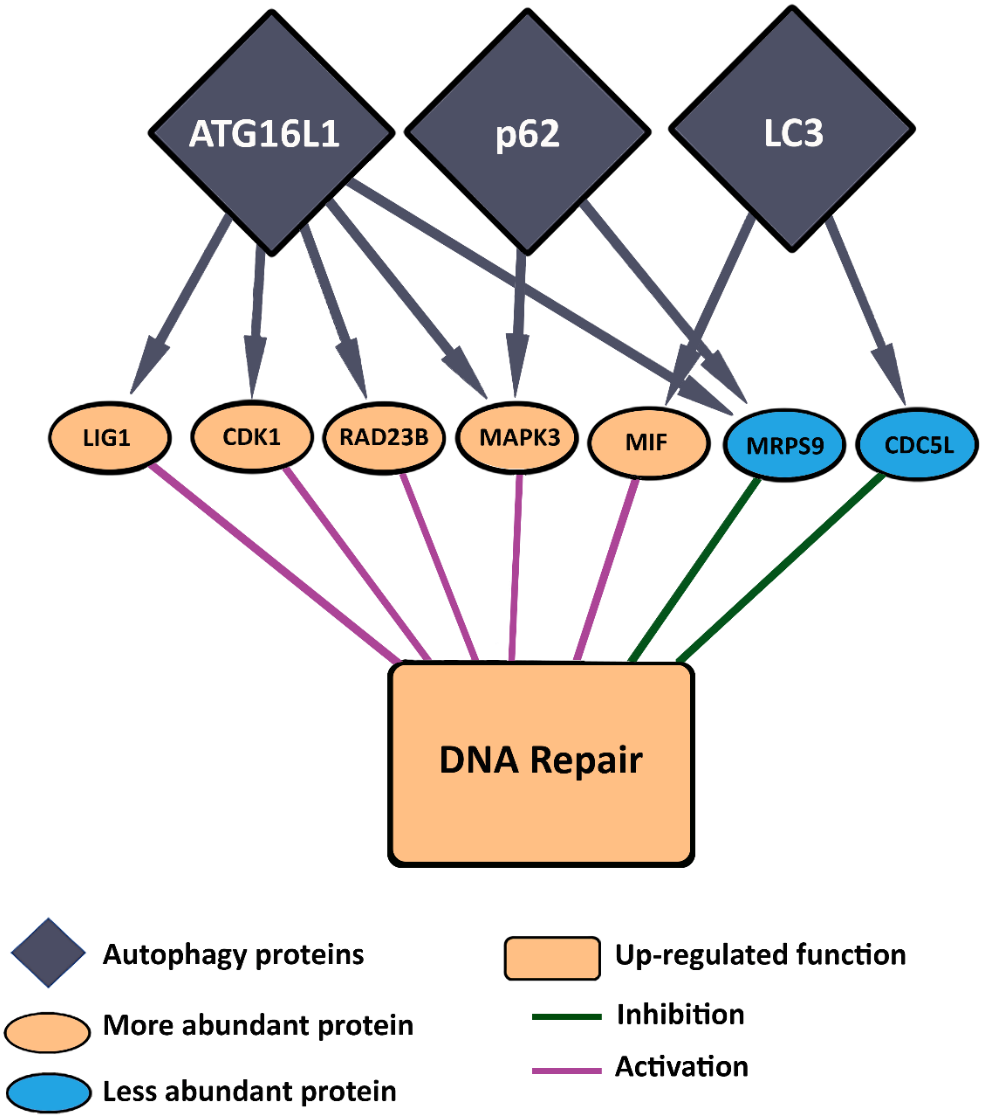

Figure S2. Alterations describing the modulation of DNA repair in response to the *Atg16L1*$^{\Delta IEC}$ mutation.

Disease Models & Mechanisms • Supplementary information

Table S1. Specific primers used in the study.

Genotype/Gene	Forward Primer Sequences<br>Reverse Primer Sequences	Number of qPCR cycles
atg16l1 $^{Fl/+}$	CTGAACAGTTAAGTTCCTAG<br>CCAAGAGACACTGACATAGG	N/A
atg16l1 $^{Fl/- Vil-Cre}$	GACGGAAATCCATCGCTCGACCAG<br>GACATGTTCAGGGATCGCCAGGCG	N/A
Villin	GTGGAATGGGCCAGAGAGT<br>CATAATTGCCATCAGCTGTGG	20
cd24	TTGCTGCTTCTGGCACTGC<br>GGAGACCAGCTGTGGACTGC	20
chromogranin A	CCAGTTCCCACTTCCATGC<br>CCTTCAGACGGCAGAGCTTC	27
lgr5	ATACCGGAGCGAGCGTTC<br>TGGCAGTTCCTGTCAAGTGA	25
muc2	GTTGCTCAATGAGATGGAGGT<br>AAGCTGCATGACTGGAAGC	20
β-actin	GAGGCCCCCCTGAACCCTAAG<br>GAACCGCTCGTTGCCAATAG	20

Disease Models & Mechanisms • Supplementary information

Table S2. Discarded biological process terms not related to intestinal functions.

Click here to Download Table S2

Table S3. Effect of the *Atg16L1*$^{\Delta IEC}$ mutation on protein abundances in normal organoid control experiment.

Click here to Download Table S3

Table S4. Effect of the *Atg16L1*$^{\Delta IEC}$ mutation on protein abundances in Paneth cell-enriched organoid control experiment.

Click here to Download Table S4

Table S5. Post-processed results from the measurements comparing the proteomic profiles of *Atg16L1*$^{\Delta IEC}$ and WT Paneth cell-enriched organoids.

Click here to Download Table S5

Table S6. Human orthologs of the mouse proteins with altered abundances.

Click here to Download Table S6

Table S7. Evidence suggesting the targeting of proteins with altered abundances by the autophagy proteins (p62, LC3, ATG16L1).

Click here to Download Table S7

Table S8. Summary of abundance changes of proteins potentially targeted by autophagy and their assigned gene ontology terms.

Click here to Download Table S8

Table S9. Functional evidence indicating the stimulatory/inhibitory effect of the proteins with altered abundances on their corresponding processes.

Click here to Download Table S9

Table S10. The aggregated trends of the gene ontology terms corresponding to the altered proteins in Paneth cells upon the deletion of *Atg16l1*.

Click here to Download Table S10

Table S11. Functional processes and their aggregated trends in Paneth cells upon the deletion of *Atg16l1*.

Click here to Download Table S11

Table S12. Overlapping functional proteins and their FC value in proteomics and transcriptomics studies

Click here to Download Table S12

Table S13. Exocytotic proteins found to be differentially abundant upon autophagy impairment.

Gene symbol	Trend in alteration (absolute fold change)	Activator/Inhibitor	Function	Reference
ANXA1	UP (3.17)	Inhibitor	Inhibitor of hormone exocytosis	McArthur et al, 2009
CDK1	UP (2.96)	Inhibitor	Inhibits the assembly of the GM130-p115-giantin tether and thus the fusion of COPI vesicles	Wang et al, 2008
GDI1	UP (2.58)	Inhibitor	Recycling of proteins from their target membranes back to their vesicular pools	Garrett et al, 1994
GABARAPL2	UP (3.5)	Activator	LC3 (containing GABARAPL-2 protein) contributes to the envelopment and exocytosis of viruses during lytic infection	Cadwell and Debnath, 2017
TMED3	DOWN (3.42)	Activator	Bind to both COPI and COPII proteins and likely function in anterograde and retrograde transport between the ER and the Golgi	Jerome-Majewska et al, 2010
GORASP1	DOWN (2.48)	Activator	COPII vesicle coating, ER to Golgi vesicle-mediated transport	Gene Ontology
SRP14	DOWN (2.37)	Activator	Targeting secretory proteins to the rough endoplasmic reticulum membrane	Gene Ontology

Disease Models & Mechanisms • Supplementary information

Reference

1.Rodriguez A, Durán A, Selloum M, Champy M-F, Diez-Guerra FJ, Flores JM, et al. Mature-onset obesity and insulin resistance in mice deficient in the signaling adapter p62. Cell Metab. 2006 Mar;3(3):211–22.

2.Wu J, Lei G, Mei M, Tang Y, Li H. A novel C53/LZAP-interacting protein regulates stability of C53/LZAP and DDRGK domain-containing Protein 1 (DDRGK1) and modulates NF-kappaB signaling. J Biol Chem. 2010 May 14;285(20):15126–36.

3.Oláhová M, Taylor SR, Khazaipoul S, Wang J, Morgan BA, Matsumoto K, et al. A redox-sensitive peroxiredoxin that is important for longevity has tissue- and stress-specific roles in stress resistance. Proc Natl Acad Sci USA. 2008 Dec 16;105(50):19839–44.

4.Tang D, Wu D, Hirao A, Lahti JM, Liu L, Mazza B, et al. ERK activation mediates cell cycle arrest and apoptosis after DNA damage independently of p53. J Biol Chem. 2002 Apr 12;277(15):12710–7.

5.Nagumo Y, Han J, Bellila A, Isoda H, Tanaka T. Cofilin mediates tight-junction opening by redistributing actin and tight-junction proteins. Biochem Biophys Res Commun. 2008 Dec 19;377(3):921–5.

6.Oddo M, Calandra T, Bucala R, Meylan PRA. Macrophage migration inhibitory factor reduces the growth of virulent Mycobacterium tuberculosis in human macrophages. Infect Immun. 2005 Jun;73(6):3783–6.

7.Le Goffe C, Vallette G, Charrier L, Candelon T, Bou-Hanna C, Bouhours J-F, et al. Metabolic control of resistance of human epithelial cells to H2O2 and NO stresses. Biochem J. 2002 Jun 1;364(Pt 2):349–59.

8.Bamburg JR, Bernstein BW. Roles of ADF/cofilin in actin polymerization and beyond. F1000 Biol Rep. 2010 Aug 19;2:62.

9.Zheng J, Yang X, Harrell JM, Ryzhikov S, Shim EH, Lykke-Andersen K, et al. CAND1 binds to unneddylated CUL1 and regulates the formation of SCF ubiquitin E3 ligase complex. Mol Cell. 2002 Dec;10(6):1519–26.

10.Zhang H, Shi X, Hampong M, Blanis L, Pelech S. Stress-induced inhibition of ERK1 and ERK2 by direct interaction with p38 MAP kinase. J Biol Chem. 2001 Mar 9;276(10):6905–8.

11.Lue H, Thiele M, Franz J, Dahl E, Speckgens S, Leng L, et al. Macrophage migration inhibitory factor (MIF) promotes cell survival by activation of the Akt pathway and role for CSN5/JAB1 in the control of autocrine MIF activity. Oncogene. 2007 Aug 2;26(35):5046–59.

12.Lieuallen K, Pennacchio LA, Park M, Myers RM, Lennon GG. Cystatin B-deficient mice have increased expression of apoptosis and glial activation genes. Hum Mol Genet. 2001 Sep 1;10(18):1867–71.

13.Menzel O, Bekkeheien RCJ, Reymond A, Fukai N, Boye E, Kosztolanyi G, et al. Knobloch syndrome: novel mutations in COL18A1, evidence for genetic heterogeneity, and a functionally impaired polymorphism in endostatin. Hum Mutat. 2004 Jan;23(1):77–84.

14.Cadwell K, Debnath J. Beyond self-eating: The control of nonautophagic functions and signaling pathways by autophagy-related proteins. J Cell Biol. 2018 Mar 5;217(3):813–22.

15.Santamaría D, Barrière C, Cerqueira A, Hunt S, Tardy C, Newton K, et al. Cdk1 is sufficient to drive the mammalian cell cycle. Nature. 2007 Aug 16;448(7155):811–5.

16.Wang Y, Wei J-H, Bisel B, Tang D, Seemann J. Golgi cisternal unstacking stimulates COPI vesicle budding and protein transport. PLoS ONE. 2008 Feb 20;3(2):e1647.

17.Sandvig K, Pust S, Skotland T, van Deurs B. Clathrin-independent endocytosis: mechanisms and function. Curr Opin Cell Biol. 2011 Aug;23(4):413–20.

18.Perretti M, Solito E. Annexin 1 and neutrophil apoptosis. Biochem Soc Trans. 2004 Jun;32(Pt3):507–10.

19.Ira G, Pellicioli A, Balijja A, Wang X, Fiorani S, Carotenuto W, et al. DNA end resection, homologous recombination and DNA damage checkpoint activation require CDK1. Nature. 2004 Oct 21;431(7011):1011–7.

20.Hong H, Kim J, Kim J. Myosin heavy chain 10 (MYH10) is required for centriole migration during the biogenesis of primary cilia. Biochem Biophys Res Commun. 2015 May 22;461(1):180–5.

21.Loru D, Incani A, Deiana M, Corona G, Atzeri A, Melis MP, et al. Protective effect of hydroxytyrosol and tyrosol against oxidative stress in kidney cells. Toxicol Ind Health. 2009 Jun;25(4-5):301–10.

22.Patibandla PK, Tyagi N, Dean WL, Tyagi SC, Roberts AM, Lominadze D. Fibrinogen induces alterations of endothelial cell tight junction proteins. J Cell Physiol. 2009 Oct;221(1):195–203.

23.Jerome-Majewska LA, Achkar T, Luo L, Lupu F, Lacy E. The trafficking protein Tmed2/p24beta(1) is required for morphogenesis of the mouse embryo and placenta. Dev Biol. 2010 May 1;341(1):154–66.

24.He H, Li J, Weng S, Li M, Yu Y. S100A11: diverse function and pathology corresponding to different target proteins. Cell Biochem Biophys. 2009 Aug 1;55(3):117–26.

25.Yablonski D, Kane LP, Qian D, Weiss A. A Nck-Pak1 signaling module is required for T-cell receptor-mediated activation of NFAT, but not of JNK. EMBO J. 1998 Oct 1;17(19):5647–57.

26.Pridgeon JW, Geetha T, Wooten MW. A Method to Identify p62's UBA Domain Interacting Proteins. Biol Proced Online. 2003 Dec 12;5:228–37.

27.Ji J, Zhao L, Wang X, Zhou C, Ding F, Su L, et al. Differential expression of S100 gene family in human esophageal squamous cell carcinoma. J Cancer Res Clin Oncol. 2004 Aug;130(8):480–6.

28.Werling D, Jungi TW. TOLL-like receptors linking innate and adaptive immune response. Vet Immunol Immunopathol. 2003 Jan 10;91(1):1–12.

29.Wu C, Keivens VM, O'Toole TE, McDonald JA, Ginsberg MH. Integrin activation and cytoskeletal interaction are essential for the assembly of a fibronectin matrix. Cell. 1995 Dec 1;83(5):715–24.

30.Thullberg M, Gad A, Beeser A, Chernoff J, Strömblad S. The kinase-inhibitory domain of p21-activated kinase 1 (PAK1) inhibits cell cycle progression independent of PAK1 kinase activity. Oncogene. 2007 Mar 15;26(12):1820–8.

31.Lan B, Chen P, Jiri M, He N, Feng T, Liu K, et al. WDR1 and CLNK gene polymorphisms correlate with serum glucose and high-density lipoprotein levels in Tibetan gout patients. Rheumatol Int. 2016 Mar;36(3):405–12.

32.Shao J, Welch WJ, Diprospero NA, Diamond MI. Phosphorylation of profilin by ROCK1 regulates polyglutamine aggregation. Mol Cell Biol. 2008 Sep;28(17):5196–208.

33.Zhao ZS, Manser E, Loo TH, Lim L. Coupling of PAK-interacting exchange factor PIX to GIT1 promotes focal complex disassembly. Mol Cell Biol. 2000 Sep;20(17):6354–63.

34.McArthur S, Yazid S, Christian H, Sirha R, Flower R, Buckingham J, et al. Annexin A1 regulates hormone exocytosis through a mechanism involving actin reorganization. FASEB J. 2009 Nov;23(11):4000–10.

35.Ohira K, Homma KJ, Hirai H, Nakamura S, Hayashi M. TrkB-T1 regulates the RhoA signaling and actin cytoskeleton in glioma cells. Biochem Biophys Res Commun. 2006 Apr 14;342(3):867–74.

36.Hartwell LH, Weinert TA. Checkpoints: controls that ensure the order of cell cycle events. Science. 1989 Nov 3;246(4930):629–34.

37.Hulkko SM, Wakui H, Zilliacus J. The pro-apoptotic protein death-associated protein 3 (DAP3) interacts with the glucocorticoid receptor and affects the receptor function. Biochem J. 2000 Aug 1;349 Pt 3:885–93.

38.Schauber C, Chen L, Tongaonkar P, Vega I, Lambertson D, Potts W, et al. Rad23 links DNA repair to the ubiquitin/proteasome pathway. Nature. 1998 Feb 12;391(6668):715–8.

39.Woolner S, O'Brien LL, Wiese C, Bement WM. Myosin-10 and actin filaments are essential for mitotic spindle function. J Cell Biol. 2008 Jul 14;182(1):77–88.

40.Witke W. The role of profilin complexes in cell motility and other cellular processes. Trends Cell Biol. 2004 Aug;14(8):461–9.

41.Samstag Y, John I, Wabnitz GH. Cofilin: a redox sensitive mediator of actin dynamics during T-cell activation and migration. Immunol Rev. 2013 Nov;256(1):30–47.

42.Arcone R, Arpaia G, Ruoppolo M, Malorni A, Pucci P, Marino G, et al. Structural characterization of a biologically active human lipocortin 1 expressed in Escherichia coli. Eur J Biochem. 1993 Jan 15;211(1-2):347–55.

43.Chun K-H, Araki K, Jee Y, Lee D-H, Oh B-C, Huang H, et al. Regulation of glucose transport by ROCK1 differs from that of ROCK2 and is controlled by actin polymerization. Endocrinology. 2012 Apr;153(4):1649–62.

44.Chen Y, Hennessy KM, Botstein D, Tye BK. CDC46/MCM5, a yeast protein whose subcellular localization is cell cycle-regulated, is involved in DNA replication at autonomously replicating sequences. Proc Natl Acad Sci USA. 1992 Nov 1;89(21):10459–63.

45.Baldin V, Cans C, Knibiehler M, Ducommun B. Phosphorylation of human CDC25B phosphatase by CDK1-cyclin A triggers its proteasome-dependent degradation. J Biol Chem. 1997 Dec 26;272(52):32731–4.

46.Yun X, Wu Y, Yao L, Zong H, Hong Y, Jiang J, et al. CDK11(p58) protein kinase activity is associated with Bcl-2 down-regulation in pro-apoptosis pathway. Mol Cell Biochem. 2007 Oct;304(1-2):213–8.

47.Chikamori K, Hill JE, Grabowski DR, Zarkhin E, Grozav AG, Vaziri SAJ, et al. Downregulation of topoisomerase IIbeta in myeloid leukemia cell lines leads to activation of apoptosis following all-trans retinoic acid-induced differentiation/growth arrest. Leukemia. 2006 Oct;20(10):1809–18.

48.Alldridge LC, Bryant CE. Annexin 1 regulates cell proliferation by disruption of cell morphology and inhibition of cyclin D1 expression through sustained activation of the ERK1/2 MAPK signal. Exp Cell Res. 2003 Oct 15;290(1):93–107.

49.Na B-R, Jun C-D. TAGLN2-mediated actin stabilization at the immunological synapse: implication for cytotoxic T cell control of target cells. BMB Rep. 2015 Jul;48(7):369–70.

50.Fujibuchi T, Abe Y, Takeuchi T, Imai Y, Kamei Y, Murase R, et al. AIP1/WDR1 supports mitotic cell rounding. Biochem Biophys Res Commun. 2005 Feb 4;327(1):268–75.

51.Woodford MR, Dunn DM, Blanden AR, Capriotti D, Loiselle D, Prodromou C, et al. The FNIP co-chaperones decelerate the Hsp90 chaperone cycle and enhance drug binding. Nat Commun. 2016 Jun 29;7:12037.

52.Wang S, Mo Y, Midorikawa K, Zhang Z, Huang G, Ma N, et al. The potent tumor suppressor miR-497 inhibits cancer phenotypes in nasopharyngeal carcinoma by targeting ANLN and HSPA4L. Oncotarget. 2015 Nov 3;6(34):35893–907.

53.Lechuga S, Baranwal S, Ivanov AI. Actin-interacting protein 1 controls assembly and permeability of intestinal epithelial apical junctions. Am J Physiol Gastrointest Liver Physiol. 2015 May 1;308(9):G745–56.

54.Pankiv S, Clausen TH, Lamark T, Brech A, Bruun J-A, Outzen H, et al. p62/SQSTM1 binds directly to Atg8/LC3 to facilitate degradation of ubiquitinated protein aggregates by autophagy. J Biol Chem. 2007 Aug 17;282(33):24131–45.

55.Candas D, Fan M, Nantajit D, Vaughan AT, Murley JS, Woloschak GE, et al. CyclinB1/Cdk1 phosphorylates mitochondrial antioxidant MnSOD in cell adaptive response to radiation stress. J Mol Cell Biol. 2013 Jun;5(3):166–75.

56.Adachi T, Sakurai T, Kashida H, Mine H, Hagiwara S, Matsui S, et al. Involvement of heat shock protein a4/apg-2 in refractory inflammatory bowel disease. Inflamm Bowel Dis. 2015 Jan;21(1):31–9.

57.Sato N, Maeda M, Sugiyama M, Ito S, Hyodo T, Masuda A, et al. Inhibition of SNW1 association with spliceosomal proteins promotes apoptosis in breast cancer cells. Cancer Med. 2015 Feb;4(2):268–77.

58.Bendix I, Pfueller CF, Leuenberger T, Glezeva N, Siffrin V, Müller Y, et al. MAPK3 deficiency drives autoimmunity via DC arming. Eur J Immunol. 2010 May;40(5):1486–95.

59.Zhang H, Ghai P, Wu H, Wang C, Field J, Zhou G-L. Mammalian adenylyl cyclase-associated protein 1 (CAP1) regulates cofilin function, the actin cytoskeleton, and cell adhesion. J Biol Chem. 2013 Jul 19;288(29):20966–77.

60.Ulrich HD, Walden H. Ubiquitin signalling in DNA replication and repair. Nat Rev Mol Cell Biol. 2010 Jun 16;11(7):479–89.

61.Kümper S, Mardakheh FK, McCarthy A, Yeo M, Stamp GW, Paul A, et al. Rho-associated kinase (ROCK) function is essential for cell cycle progression, senescence and tumorigenesis. elife. 2016 Jan 14;5:e12994.

62.Chang S, Kim JH, Shin J. p62 forms a ternary complex with PKCzeta and PAR-4 and antagonizes PAR-4-induced PKCzeta inhibition. FEBS Lett. 2002 Jan 2;510(1-2):57–61.

63.Pestov DG, Strezoska Z, Lau LF. Evidence of p53-dependent cross-talk between ribosome biogenesis and the cell cycle: effects of nucleolar protein Bop1 on G(1)/S transition. Mol Cell Biol. 2001 Jul;21(13):4246–55.

64.Schwerk C, Prasad J, Degenhardt K, Erdjument-Bromage H, White E, Tempst P, et al. ASAP, a novel protein complex involved in RNA processing and apoptosis. Mol Cell Biol. 2003 Apr;23(8):2981–90.

65.Knezevic N, Roy A, Timblin B, Konstantoulaki M, Sharma T, Malik AB, et al. GDI-1 phosphorylation switch at serine 96 induces RhoA activation and increased endothelial permeability. Mol Cell Biol. 2007 Sep;27(18):6323–33.

66.Frost JA, Khokhlatchev A, Stippec S, White MA, Cobb MH. Differential effects of PAK1-activating mutations reveal activity-dependent and -independent effects on cytoskeletal regulation. J Biol Chem. 1998 Oct 23;273(43):28191–8.

67.Van Hemert MJ, Steensma HY, van Heusden GP. 14-3-3 proteins: key regulators of cell division, signalling and apoptosis. Bioessays. 2001 Oct;23(10):936–46.

68.Wilmink GJ, Roth CL, Ibey BL, Ketchum N, Bernhard J, Cerna CZ, et al. Identification of microRNAs associated with hyperthermia-induced cellular stress response. Cell Stress Chaperones. 2010 Nov;15(6):1027–38.

69.Amson R, Pece S, Marine J-C, Di Fiore PP, Telerman A. TPT1/ TCTP-regulated pathways in phenotypic reprogramming. Trends Cell Biol. 2013 Jan;23(1):37–46.

70.Denz A, Pilarsky C, Muth D, Rückert F, Saeger H-D, Grützmann R. Inhibition of MIF leads to cell cycle arrest and apoptosis in pancreatic cancer cells. J Surg Res. 2010 May 1;160(1):29–34.

71.Bertling E, Hotulainen P, Mattila PK, Matilainen T, Salminen M, Lappalainen P. Cyclase-associated protein 1 (CAP1) promotes cofilin-induced actin dynamics in mammalian nonmuscle cells. Mol Biol Cell. 2004 May;15(5):2324–34.

72.Grankvist N, Amable L, Honkanen RE, Sjöholm A, Ortsäter H. Serine/threonine protein phosphatase 5 regulates glucose homeostasis in vivo and apoptosis signalling in mouse pancreatic islets and clonal MIN6 cells. Diabetologia. 2012 Jul;55(7):2005–15.

73.Garrett MD, Zahner JE, Cheney CM, Novick PJ. GDI1 encodes a GDP dissociation inhibitor that plays an essential role in the yeast secretory pathway. EMBO J. 1994 Apr 1;13(7):1718–28.

74.Zhang Z, Morla AO, Vuori K, Bauer JS, Juliano RL, Ruoslahti E. The alpha v beta 1 integrin functions as a fibronectin receptor but does not support fibronectin matrix assembly and cell migration on fibronectin. J Cell Biol. 1993 Jul;122(1):235–42.

75.Susin SA, Lorenzo HK, Zamzami N, Marzo I, Snow BE, Brothers GM, et al. Molecular characterization of mitochondrial apoptosis-inducing factor. Nature. 1999 Feb 4;397(6718):441–6.

76.Ewing RM, Chu P, Elisma F, Li H, Taylor P, Climie S, et al. Large-scale mapping of human protein-protein interactions by mass spectrometry. Mol Syst Biol. 2007 Mar 13;3:89.

77.Berger KL, Cooper JD, Heaton NS, Yoon R, Oakland TE, Jordan TX, et al. Roles for endocytic trafficking and phosphatidylinositol 4-kinase III alpha in

hepatitis C virus replication. Proc Natl Acad Sci USA. 2009 May 5;106(18):7577–82.

78.Behrends C, Sowa ME, Gygi SP, Harper JW. Network organization of the human autophagy system. Nature. 2010 Jul 1;466(7302):68–76.

79.Galvez T, Gilleron J, Zerial M, O'Sullivan GA. SnapShot: Mammalian Rab proteins in endocytic trafficking. Cell. 2012 Sep 28;151(1):234–234.e2.
